# Melt Electrowriting of Polyhydroxyalkanoates for Enzymatically Degradable Scaffolds

**DOI:** 10.1002/adhm.202401504

**Published:** 2024-11-12

**Authors:** Magdalena Z. Gładysz, Didi Ubels, Marcus Koch, Armin Amirsadeghi, Frederique Alleblas, Sander van Vliet, Marleen Kamperman, Jeroen Siebring, Anika Nagelkerke, Małgorzata K. Włodarczyk‐Biegun

**Affiliations:** ^1^ Polymer Science Zernike Institute for Advanced Materials University of Groningen Nijenborgh 4 Groningen 9747 AG The Netherlands; ^2^ Pharmaceutical Analysis Groningen Research Institute of Pharmacy University of Groningen P.O. Box 196, XB20 Groningen 9700 AD The Netherlands; ^3^ INM‐Leibniz Institute for New Materials Campus D2 2 66123 Saarbrücken Germany; ^4^ Bioconversion and Fermentation Technology Research Centre Biobased Economy Hanze University of Applied Sciences Zernikeplein 11 Groningen 9747 AS The Netherlands; ^5^ Biotechnology Centre Silesian University of Technology Krzywoustego 8 Gliwice 44‐100 Poland

**Keywords:** 3D printing, biodegradable polymers, P34HB, poly(3‐hydroxybutyrate‐*co*‐3‐hydroxyvalerate), poly(3‐hydroxybutyrate‐*co*‐4‐hydroxybutyrate

## Abstract

Melt electrowriting (MEW) enables precise scaffold fabrication for biomedical applications. With a limited number of processable materials with short and tunable degradation times, polyhydroxyalkanoates (PHAs) present an interesting option. Here, poly(3‐hydroxybutyrate‐co‐3‐hydroxyvalerate) (PHBV) and a blend of PHBV and poly(3‐hydroxybutyrate‐co‐4‐hydroxybutyrate) (PHBV+P34HB) are successfully melt electrowritten into scaffolds with various architectures. PHBV+P34HB exhibits greater thermal stability, making it a superior printing material compared to PHBV in MEW. The PHBV+P34HB scaffolds subjected to enzymatic degradation show tunable degradation times, governed by enzyme dilution, incubation time, and scaffold surface area. PHBV+P34HB scaffolds seeded with human dermal fibroblasts (HDFs), demonstrate enhanced cell adherence, proliferation, and spreading. The HDFs, when exposed to the enzyme solutions and enzymatic degradation residues, show good viability and proliferation rates. Additionally, HDFs grown on enzymatically pre‐incubated scaffolds do not show any difference in behavior compared those grown on control scaffolds. It is concluded that PHAs, as biobased materials with enzymatically tunable degradability rates, are an important addition to the already limited set of materials available for MEW technology.

## Graphical abstract

Melt electrowriting (MEW) enables fabrication of high‐resolution scaffolds for tissue engineering, yet few printable materials are available. Polyhydroxyalkanoates (PHAs), like PHBV and PHBV+P34HB, excel as biobased, biodegradable, and well‐printable materials, with PHBV+P34HB exhibiting superior thermal stability. The enzymatically degradable scaffolds allow adjustable degradation rates and reveal biocompatibility with human dermal fibroblasts. PHAs stand as a powerful alternative for gold‐standard poly(ε‐caprolactone).

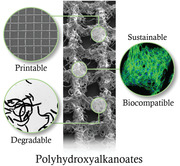

## 1 Introduction

3D printing is an attractive method to produce in vitro testing platforms and implants.^[^ ^1^ ^]^ Among scaffold fabrication techniques that utilize polymers for biomedical applications, the most commonly used are fused deposition modeling (FDM), selective laser sintering (SLS), stereolithography (SLA), direct ink writing (DIW), electrospinning, and, most recently, melt electrowriting (MEW).^[^ ^2^, ^3^, ^4^ ^]^ MEW is gaining increasing interest, as it allows for the deposition of well‐aligned polymeric microfibers with high control over the printed architecture while maintaining a high degree of porosity.^[^ ^5^ ^]^ High porosity is beneficial for cell penetration and migration, as well as the introduction of media or other substances to the scaffold, such as enzyme solutions or decellularized extracellular matrix (ECM). MEW scaffolds have been shown to facilitate cell proliferation and migration, as they provide excellent support for cell growth, polarization, and guidance.^[^ ^6^, ^7^ ^]^

Due to its recent emergence and specific requirements for a successful printing process, materials that can be used for MEW are limited. The most widely used polymer is medical grade poly(ε‐caprolactone) (PCL). It is well‐printable and approved by the United States Food and Drug Administration. However, it has a relatively long degradation time in vivo, estimated at two to three years.^[^ ^8^ ^]^ The PCL degradation rate has been assessed by a number of researchers, and a significant decrease in the number‐average molecular weight (M_n_) over time was observed, as presented in **Table** **1**.^[^ ^9^ ^]^

**Table 1:** Reported PCL degradation times presented through the decrease in the M_n_. All of the experiments were performed at 37 °C.

Study	Initial M_n_ [kDa]	Post‐degradation M_n_ kDa]	Test Environment	Time
Yavuz et al.^[^ ^10^ ^]^	30	21	Cell culture medium	30 days
	30	20	Cell culture medium	60 days
Larranaga et al.^[^ ^11^ ^]^	78	70	Phosphate‐buffered saline (PBS)	132 days (4.5 months)
Domingos et al.^[^ ^12^ ^]^	34	32	PBS	6 months
	34	33	Simulated body fluid (SBF)	6 months
Little et al.^[^ ^13^ ^]^	63	60	PBS	7 months
Coombes et al.^[^ ^14^ ^]^	78	40	PBS	45 months

In timeframes ranging from 30 days to 45 months (over 3 years), the pure PCL material exhibits degradation by showing variable ranges in the decrease of the M_n_. The degradation rate of PCL can be further accelerated by modifications to the material or additional treatment. Shen et al. reported that an electrospun PCL membrane after 48 h of potassium permanganate treatment, lost 25% of its initial mass when incubated in SBF at 37 °C for 12 weeks.^[^ ^15^ ^]^ Zhou et al. reported that PCL scaffolds etched with sodium hydroxide for 30 min and immersed in PBS for 15 days, showed a mass loss of ≈53% compared to the initial value.^[^ ^16^ ^]^ The degradation rate can also be enhanced through the incubation with enzymes such as Pseudomonas Lipase, which resulted in a 20% weight loss over 14 weeks.^[^ ^17^ ^]^ In all of the experiments, the degradation times of pure PCL substrates ranged from weeks to months. However, it should be noted that the degradation times of scaffolds depend heavily on their size and (micro)structure. Another solution is the fabrication of blends, such as polyglycolic/polycaprolactone (PGA/PCL) blend, which was fully degraded in PBS at 37 °C within 2–10 days.^[^ ^18^ ^]^

Depending on the application, shorter and tunable degradation times might be needed for tissue engineering purposes, to match the regeneration time of specific tissues. Minimizing the necessary time that the scaffold exists in the body renders implants with minimal foreign body reaction after placing them in the patient's body superior. Therefore, other potentially degradable or dissolvable polymers have been studied with the MEW technique, such as PCL blends with poly(hydroxymethylglycolide‐co‐ε‐caprolactone), poly(2‐ethyl‐2‐oxazoline), poly(2‐ethyl‐2‐oxazine), and poly(l‐lactide‐co‐acryloyl carbonate).^[^ ^19^ ^]^ Scaffolds made out of PCL blended with poly(hydroxymethylglycolide‐co‐ε‐caprolactone) have been developed for cardiac tissue by Castilho et al. (2017).^[^ ^20^ ^]^ This system potentially allows for controlled degradation times, although detailed studies were not provided. As an indication, Seyednejad et al. (2012) showed that such blends can lose over 60% of their initial weight three months after implantation.^[^ ^21^ ^]^ Similarly, for other polymers, such as poly(2‐ethyl‐2‐oxazoline), poly(2‐ethyl‐2‐oxazine), and poly(l‐lactide‐co‐acryloyl carbonate), precise information about degradation or dissolution times is still missing.^[^ ^22^, ^23^, ^24^ ^]^ Therefore, there is a clear need for a material with tunable degradation times, shorter than years, that can be used for MEW.

A promising candidate for bridging this gap is a group of aliphatic polyesters called polyhydroxyalkanoates (PHAs). PHAs are synthesized by microorganisms, as carbon storage to function as an energy reservoir, when subjected to stress conditions such as a carbon surplus or lack of oxygen.^[^ ^25^, ^26^ ^]^ This can be seen as a new approach to green chemistry as polymers are produced by bacteria without the use of additional solvents and with minimal waste. After extraction from microbial cultures, PHAs can be utilized for various purposes, such as filaments for 3D printing, packaging, films, and the production of cosmetics.^[^ ^27^ ^]^ Importantly, PHAs can be degraded with a PHA depolymerase enzyme when needed.^[^ ^28^ ^]^ Commonly encountered PHAs in biomedical science are poly(3‐hydroxybutyrate) (PHB), poly(3‐hydroxyvalerate) (PHV), poly(3‐hydroxybutyrate‐*co*‐3‐hydroxyvalerate) (PHBV), and poly(3‐hydroxybutyrate‐*co*‐4‐hydroxybutyrate) (P34HB).^[^ ^29^, ^30^, ^31^, ^32^, ^33^, ^34^ ^]^ However, there are over 150 identified PHA monomeric units, which creates ample opportunities to produce other PHAs, with tailor‐made properties, such as specific crystallinity, flexibility, or melting temperature without compromising biodegradability.^[^ ^4^, ^35^ ^]^ It is important to highlight that the primary factor contributing to PCL degradation is non‐enzymatic random chain scission, which occurs via both acid‐ and base‐catalyzed ester hydrolysis. In contrast, PHAs undergo minimal auto‐hydrolysis, with random chain scission playing a negligible role in their degradation. Instead, PHAs degrade mainly through surface erosion, making the degradation process more controllable.^[^ ^9^ ^]^

In this paper, by combining new materials and emerging printing technology, MEW‐processed PHA scaffolds with unique properties are investigated. They promote cell adhesion and proliferation and can be removed on demand via enzymatic degradation applied before implantation. It is shown that PHBV and its blend with P34HB (referred to hereafter as PHBV+P34HB) can be 3D‐printed via MEW to obtain multilayered architectures with various fiber orientations. These PHA scaffolds exhibit higher Young's moduli than melt electrowritten PCL scaffolds of similar design but are more brittle, especially when printed toward the end of a printing cycle, suggesting material degradation at elevated temperatures. The enzymatic degradability of PHBV+P34HB, as well as its mechanical properties and thermal stability during printing, are determined to be superior to those of PHBV. Further, PHBV+P34HB filaments and scaffolds are subjected to various PHA depolymerase solutions, after which their influence on the polymeric surface, structure, and scaffold integrity is observed over time. Finally, different enzyme concentrations and incubation times are successfully used to tune the degradation time. Conventional rigid polymer scaffolds can hinder the functionality of flexible tissues such as skin and cartilage, often necessitating removal. In poorly perfused tissues, the in vivo degradation of scaffolds remains uncertain. In this context, PHAs present a promising solution, providing 3D structures that support cell growth and are enzymatically degradable in a controlled manner prior to implantation. To explore applications of the proposed scaffolds for the development of skin models, cell culture studies are conducted using primary human dermal fibroblasts (HDFs), and the potential for future smart implants is highlighted.

## 2 Results and Discussion

### 2.1 Melt Electrowriting of Scaffolds

Two PHAs, namely PHBV and PHBV+P34HB, were explored as potential printing materials for MEW. A temperature of 180 °C was used for the printing of both materials, as this was the lowest temperature at which these polymers flow and form a stable jet. To slow down the thermal degradation during printing, the printing cartridge with the polymer inside was subjected to a nitrogen purge. PHBV was found to be printable after 20 min of pre‐heating at 180 °C in the printer cartridge. The scaffolds printed at the beginning of the cycle faithfully represented designed geometries with straight fibers (Figure S1A, Supporting Information). Upon heating for longer than 30 min in total, a “whipping” effect was observed (Figure S1B,C, Supporting Information). For PCL, we correlated the “whipping” with the increase in electric charges within the fibers, during printing below the critical translation speed, causing the strand to coil due to the repulsion of the internal charges on the strand. This resulted in the formation of irregular shapes upon deposition. However, “whipping” effect in PHBV is rather related to the material's decrease in viscosity caused by thermal degradation and the molecular weight decrease caused by the prolonged heating in the cartridge (Figure S2, Supporting Information). The whipping was overcome by increasing the printing speed with a subsequent decrease in voltage to accommodate a more stable jet formation (Figure S1D,E, Supporting Information). Increasing the inter‐fiber distance in the final scaffolds was also found to limit the whipping. Finally, after a prolonged residence time in the heated cartridge (over an hour), the thermal degradation of the material became even more pronounced (Figure S1F, Supporting Information), as indicated by the irregularities within the fibers (Figure S3, Supporting Information), increased brittleness of the scaffolds while handling, and a color change, from off‐white to yellow. Overall, the printability window for PHBV was found to be between 20 and 80–90 min. After that time point, the material must be taken out of the cartridge and replaced.

In contrast, PHBV+P34HB was printable only after 40–45 min of pre‐heating. Compared with PHBV, the material flow was slower, which allowed printing at a lower speed. The first prints of the cycle were typically printed in straight lines (Figure S4A, Supporting Information). However, after 50–65 min of heating, whipping occurred (Figure S4B,C, Supporting Information), which required adjustments to the printing parameters for further deposition of straight fibers (Figure S4D, Supporting Information). PHBV+P34HB also allowed for the printing of more layers than with PHBV (six instead of four) under the same conditions of speed, voltage, and pressure. The printability time window for PHBV+P34HB was longer than for PHBV, ranging from 40 to 160 min. After 160 min, the material needed to be replaced, as it was no longer possible to extrude it.

**Figure 1:**
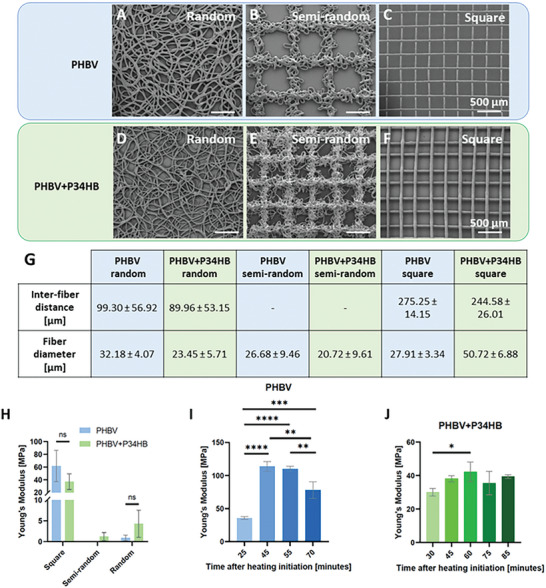
A–F) Scanning electron microscopy (SEM) images of three fabricated architectures (random, semi‐random, and square, respectively) printed from A–C) PHBV, and D–F) PHBV+P34HB. Scale bars: 500 µm. G) Table with measured fiber diameters and inter‐fiber distances for each type of printed scaffold. The values refer to the calculated average distance/diameter ± SD, *n* = 22. The inter‐fiber distances of the semi‐random scaffolds are not given, due to their bimodality. H–J) Mechanical properties of melt electrowritten PHBV and PHBV+P34HB scaffolds as measured by uniaxial tensile testing. H) Young's Modulus of the scaffolds with three different geometries; I) PHBV square scaffolds printed at different time points after heating initiation; J) PHBV+P34HB square scaffolds printed at different time points after heating initiation. Data are presented as mean ± SD, *n* = 6. Significant statistical difference: *p ≤ 0.05; **p ≤ 0.01; ***p ≤ 0.001; ****p ≤ 0.0001, *ns* = not significant.

Overall, both polymers allowed for printability of various architectures, as shown in **Figure** **1** (printing parameters shown in Table S1, Supporting Information). The smallest inter‐fiber distances printed for the square mesh were ≈250 µm, with the PHBV+P34HB fiber diameter being notably larger than the PHBV fibers (50 µm in comparison with 28 µm, respectively), as indicated in Figure 1G. Both inter‐fiber and diameter values are comparable with the MEW prints that can be obtained using gold standard PCL.^[^ ^36^ ^]^ This shows that MEW can be utilized to achieve higher‐resolution prints than the standard printing techniques employed to create PHA scaffolds, such as FDM or SLS.^[^ ^4^ ^]^

### 2.2 Mechanical Properties

To shed light on the mechanical properties of the printed PHA scaffolds, uniaxial tensile tests were performed on three scaffold types (random, semi‐random, and square with 300 µm inter‐fiber distances) printed with PHBV+P34HB and random and square designs printed with PHBV. The semi‐random scaffolds printed with PHBV were too fragile to be measured. Young's moduli obtained for the square scaffolds made of PHBV and PHBV+P34HB (61.71 ± 24.65 MPa and 37.10 ± 12.46 MPa, respectively) were higher than values reported in the literature for similar scaffolds made of PCL (usually less than 10 kPa) (Figure 1H).^[^ ^20^, ^37^ ^]^ The lower Young's moduli obtained for the PHBV+P34HB scaffolds than for the PHBV scaffolds can be attributed to the presence of the 4‐hydroxybutyrate monomer, which is known to influence the polymer's mechanical properties.^[^ ^38^ ^]^ The scaffolds printed in a randomly organized shape showed much lower Young's moduli (0.91 ± 0.064 MPa and 4.29 ± 3.25 MPa for PHBV and PHBV+P34HB respectively) and were easily stretchable under low forces compared to the square scaffolds, as fewer fibers were aligned in the direction of the force pulling on the scaffold. Additionally, some elongation of the single‐printed fibers was observed for square scaffolds. The relatively large error bars of the stiffness values suggest some variability in the samples, which can be attributed to randomly oriented fibers being stretched in a variable manner, especially for random and semi‐random scaffolds. The large error bars of the square mesh scaffolds (Figure 1H) are a result of variability in heating time before printing of the measured scaffolds. Indeed, the range of error corresponds to the ranges of Young's modulus values obtained (Figure 1I,J) when different heating times were considered.

As the thermal degradation likely influences the mechanical properties of the printed scaffolds, square scaffolds were printed using both materials at different heating times, and their mechanical properties were measured with uniaxial tensile testing (Figure 1I). It was observed that Young's moduli of PHBV scaffolds increased significantly between 25 and 45 min of printing, yet slightly decreased after 70 min. As for PHBV+P34HB, Young's moduli also increased with the printing time but to a lesser extent, with only one significant difference between 30 and 60 min of printing (Figure 1J). Considering both Young's moduli in relation to the printing time and the previously discussed printing window, PHBV+P34HB showed more favorable properties. This blend was therefore chosen for further studies. In the future, to increase the printability window, the PHA filament can be printed with the use of a filament‐based MEW device, which would decrease the time that the printing material is held in the hot cartridge.^[^ ^39^ ^]^

### 2.3 Thermal Stability

**Figure 2:**
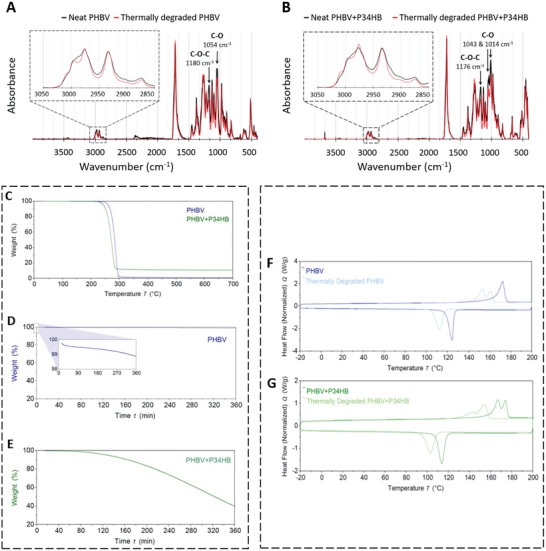
A,B) Transposed and superimposed FTIR spectra of A) PHBV, and B) PHBV+P34HB. C–E) TGA of PHBV (blue) and PHBV+P34HB (green). C) Conventional TGA of PHBV and PHBV+P34HB, heating from 20 to 700 °C; D) Long‐exposure TGA of PHBV at 180 °C; E) Long‐exposure TGA of PHBV+P34HB at 180 °C. F,G) DSC from −20 to 200 °C of neat and thermally degraded F) PHBV and G) PHBV+P34HB.

To better understand the thermal stability of the material, Fourier‐transform infrared spectroscopy (FTIR), thermal gravimetric analysis (TGA), differential scanning calorimetry (DSC), and gel permeation chromatography (GPC) were performed on the neat (unheated filament) and thermally degraded (heated for 5 h at 180 °C) material. FTIR was employed to observe the relative composition differences between the neat and thermally degraded PHBV and PHBV+P34HB (**Figure** **2**A,B). Noticeable differences in absorbance spectra between neat and thermally degraded samples were observed as a decrease in absorbance at certain wavenumbers for the thermally degraded samples. For PHBV this decrease was observed at 1180 and 1054 cm^−1^ which corresponds to C─O─C stretching and C─O stretching of an ester linkage, respectively.^[^ ^40^, ^41^ ^]^ For PHBV+P34HB, the lowering of the peaks occurred at 1176 cm^−1^, which was attributed to C─O─C stretching, and at 1043 and 1014 cm^−1^, which were assigned to C─O stretching of an ester group. These changes indicate a decrease in the overall concentration of ester groups compared to other functional groups in the thermally degraded samples. Therefore, it is likely that the thermal degradation mechanism occurs in the form of β‐elimination at the ester linkages.^[^ ^42^ ^]^ Another noteworthy difference in the FTIR spectra between neat and thermally degraded polymers was observed in the 2850 to 3050 cm^−1^ range, where more distinguishable peaks appeared ≈3000 cm^−1^ in the thermally degraded samples. These peaks were assigned to the C─H stretching and O─H stretching of carboxylic acid groups, which indicates the formation of oligomers.^[^ ^43^ ^]^

The TGA showed that the temperature at which the steepest drop in sample weight occurs for both polymers lies between 250 and 300 °C (Figure 2C). However, both polymers showed signs of thermal degradation during printing at 180 °C, which is inconsistent with these TGA findings. Therefore long‐exposure TGAs at 180 °C were conducted. PHBV showed only 1% weight loss within 6 h (Figure 2D), which was inconsistent with the observations while printing as the material had to be replaced after roughly 45 min. PHBV+P34HB showed a gradual decrease in weight with time, which started after ca. 90 min, reaching 40% of its initial weight (Figure 2E). The empirically established printing window for PHBV+P34HB was between 40 and 160 min, so this was done before the sample weight dropped below 90% (Figure 2C). The results from TGA suggest that PHBV is a better candidate for printing than PHBV+P34HB, as it is more thermally stable. Such a finding is not corroborated by the empirical finding that PHBV+P34HB had a longer printability window.

Therefore, to further investigate the thermal behavior of both polymers, DSC analyses were conducted (Figure 2F,G). In the case of neat PHBV, the observed melting temperature of 173 °C (**Table** **2**) matched previously reported melting temperatures for PHBV samples with a low concentration of 3‐hydroxyvalerate (3HV) monomer.^[^ ^44^ ^]^ This was in line with expectations as the PHBV used had a low 3HV content. The melting point of PHBV decreases when the amount of 3HV monomer increases, showing the tunability of PHAs.^[^ ^45^ ^]^ For the thermally degraded PHBV, the melting peak observed with the neat sample was separated into two melting points. The melting points for neat PHBV+P34HB were at 167 and 174 °C. These values were higher than the melting points of 127 and 146 °C, found in the literature for the P34HB.^[^ ^46^ ^]^ Therefore it was concluded that adding PHBV to P34HB elevated both of its melting points. The peaks registered for thermally degraded PHBV+P34HB had lower values (143 and 154 °C). This decline was attributed to an overall reduction in the polymer chain lengths resulting from thermal degradation in the form of β‐elimination of the ester linkages. For both thermally degraded materials, PHBV and PHBV+P34HB, the measured values of enthalpy of melting and crystallization were lower than those obtained for the neat samples (Table 2). Such an observation may stem from the fact that with the thermal degradation process, the internal energy of the polymer decreases. As the chains become smaller due to thermal degradation, there will be fewer covalent bonds present within the polymer and therefore a lower overall binding energy. The DSC results for the neat and thermally degraded materials did not display a glass transition temperature (*Tg*). While this finding diverges from some literature reports, it is not uncommon in DSC studies of polymers, where the observation of *Tg* can be elusive.^[^ ^46^, ^47^ ^]^

**Table 2:** Measured DSC and GPC values for the investigated materials. TD stands for thermally degraded material, T_m_ and T_c_ are melting and crystallization temperatures, respectively, and ΔH_m_ and ΔH_c_ are the enthalpy of melting and crystallization, respectively. M_n_ is the number‐average molecular weight, M_w_ is the weight‐average molecular weight, and Đ is polymer dispersity calculated as a ratio of M_w_ to M_n_.

Material	*T_m1_* [°C]	*T_m2_* [°C]	Δ*H_m_* [J g^−1^]	*T_c_* [°C]	Δ*H_c_* [J g^−1^]	*M_n_* [g mol^−1^]	*M_w_* [g mol^−1^]	Đ [‐]
PHBV	173	−	100	124	93	98009	179654	1.8
TD PHBV	153	161	96	112	87	9045	21601	2.4
PHBV+P34HB	167	174	62	114	59	71233	176617	2.5
TD PHBV+P34HB	143	154	55	103	52	1712	4555	2.7

For both materials, the empirically chosen printing temperature of 180 °C was above their melting points. The DSC results also confirmed the hypothesis of material thermal degradation. To lower the printing temperature, a polymer with a higher content of P34HB could be used in the future.

Finally, PHBV and PHBV+P34HB were investigated via GPC as neat and thermally degraded samples. The results indicated a decrease in molecular weight (shorter chain lengths) in the thermally degraded samples compared to the neat ones. At the same time, the dispersity (Đ) of the thermally degraded samples showed an increased value (Table 2), indicating a larger distribution of molecular weights. On the molecular weight distribution plots (Figure S5, Supporting Information), a larger shift in the direction of low molecular weights can be seen for the thermally degraded PHBV+P34HB than for PHBV, also reflected by the M_n_ and M_w_ values seen in Table 2.

### 2.4 Enzymatic Degradation

**Figure 3:**
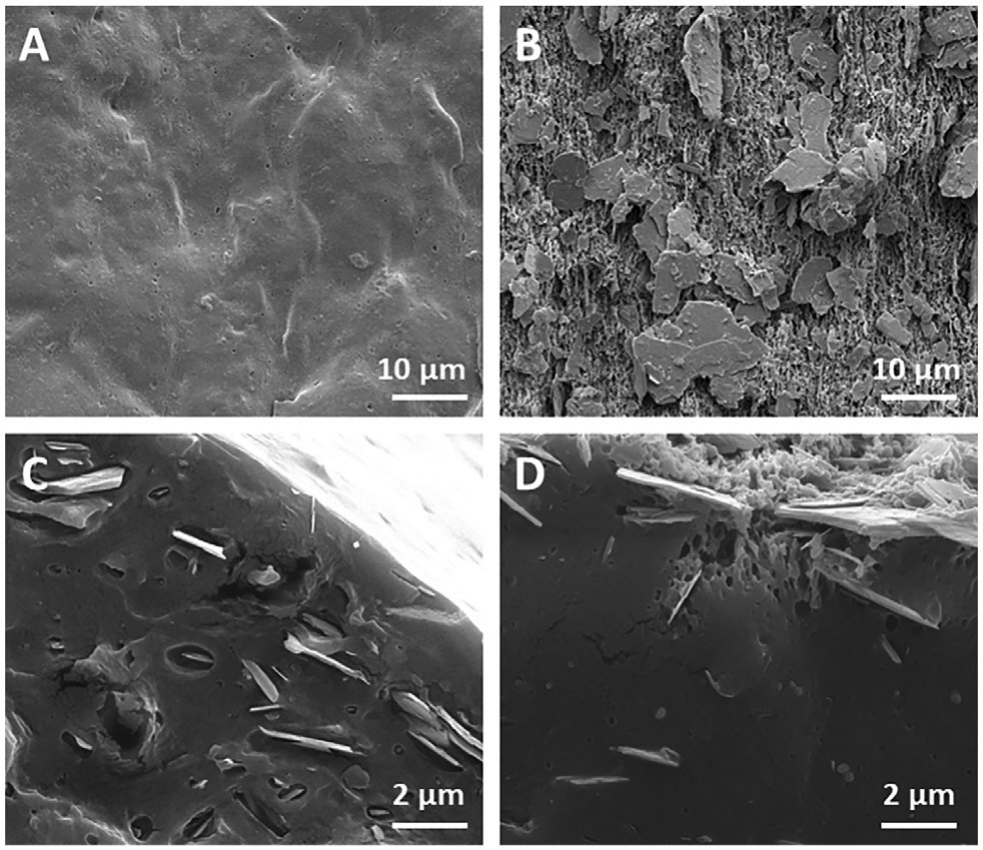
SEM imaging of A) Surface of the control PHBV+P34HB filament, B) enzymatically degraded PHBV+P34HB filament, C) cross‐section of control printed fiber, D) cross‐section of enzymatically degraded filament. Scale bars: A, B) 10 µm, C, D) 2 µm.

PHAs can be enzymatically degraded by the specific enzyme PHA depolymerase (PhaZ), which can be beneficial for applications in tissue engineering. To investigate the degradation rate of PHBV+P34HB, a single printing filament was incubated in PhaZ solution and, as a control, in enzyme elution buffer (20 mM sodium phosphate, 300 mM NaCl, 250 mM imidazole). After 2 days of incubation at room temperature in the presence of the enzyme, the solution became turbid. No changes were noted for the controls. SEM images of the samples were taken afterwards. As shown in **Figure** **3**A,B, the enzymatically degraded samples had a more uneven surface with crystal‐like flakes becoming visible. Such flakes were also seen in the bulk (center of the material) of both the enzymatically degraded and the control filaments (Figure 3C,D). Moreover, in the cross‐section of the enzymatically degraded filament, the formation of pores close to the surface was observed (Figure 3D), which was attributed to the polymer being broken down by PhaZ first at the surface and then in the bulk. It was hypothesized that by enlarging the surface area, the degradation time could be adjusted.

The enzymatic degradation process initiates when enzymes adsorb onto the surface of the PHA. This is followed by a non‐hydrolytic disruption of the polymer's structure, eventually leading to hydrolysis.^[^ ^48^ ^]^ Hydrophobic and electrostatic interactions, along with hydrogen bonding between several amino acid residues, facilitate the enzyme‐substrate interaction.^[^ ^49^ ^]^ Once the enzyme binds to the surface of the material, it disrupts the chain‐packing regions, causing structural destabilization. The process concludes with the catalysis of the hydrolysis of the ester bonds present between the PHA monomers, resulting in the release of oligomers and monomers from the main backbone. Moreover, the hydrolysis process of the ester bonds between the PHA monomers results in a pH decrease.^[^ ^50^ ^]^

We hypothesize that the enzymes introduced to the PHAs adsorb to the surface and remain attached during cell culture, despite medium changes, and begin to hydrolyze the ester bonds even after short incubation periods. These enzymes likely remain on the surface, continuing to degrade the PHAs until the pH drops too low (Figure S6, Supporting Information).

After the degradation process, a leftover residue in the form of a powder was detected, which did not enzymatically degrade. It was hypothesized that this powder originated from the crystal‐like flakes identified earlier in the PHBV+P34HB filament (Figure 3C). To determine their contents, energy‐dispersive X‐ray (EDX) combined with SEM, transmission electron microscopy (TEM), and X‐ray diffraction (XRD) tests were performed (Figures S7–S9, Supporting Information). The elements identified in the crystal flakes by EDX (Figure S7, Supporting Information) were carbon (C), oxygen (O), boron (B), nitrogen (N), magnesium (Mg), and silicon (Si). The visible gold (Au) peaks were associated with the imaging technique used (sputter‐Au) (Figure S7C,D, Supporting Information). The carbon and part of the oxygen can be attributed to the remains of the PHA building blocks, the latter elements indicate that the flakes could consist of minerals, namely talc (Mg_3_Si_4_O_10_(OH)) and boron nitride (BN). Literature has shown that talc and BN lower the crystallization barriers and increase the crystallization rate for PHB and PHBV.^[^ ^51^, ^52^ ^]^ The XRD of the leftover powder (Figure S8, Supporting Information) showed very clear diffraction peaks, attributed to the crystalline nature of the investigated sample and confirming the suspicion of minerals being present. Combined with data from EDX on the filament (Figure S7, Supporting Information) and the powder (Figure S9, Supporting Information), these findings suggest that they were indeed both composed of several mineral additives, namely BN, talc, and titanium oxide (TiO_2_). Investigation via TEM was used to determine that TiO_2_ is present in the form of nanoparticles (Figure S9B, Supporting Information). These findings therefore suggested that the printed material was not a pure PHBV+P34HB but a composition of it together with mineral additives included at the manufacturing stage. The addition of these minerals to polymers such as PHAs causes a higher crystallization rate, as it directly affects the nucleation process and the suppression of the Tg, which explains why the DSC graphs for the PHAs did not exhibit a Tg (Figure 2).^[^ ^53^, ^54^ ^]^

**Figure 4:**
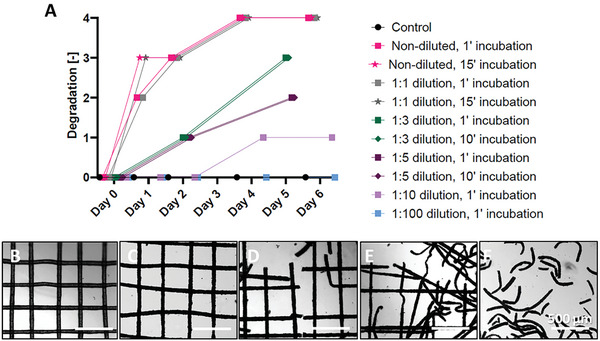
A) Conceptual graph showing the enzymatic degradation rate of MEW‐printed PHBV+P34HB scaffolds depending on enzyme dilution in Dulbecco's Modified Eagle Medium (DMEM) medium and on the time of scaffold incubation in the enzyme solution. The degradation extent, based on the brightfield microscopy images, of the investigated scaffold: B) intact scaffold: grade 0; C) grade 1; D) grade 2; E) grade 3; F) grade 4. Scale bars: 500 µm.

To analyze the biocompatibility of the degradation process, square scaffolds were printed with PHBV+P34HB and subjected to PhaZ solutions (**Figure** **4**). The fastest rate was observed for the non‐diluted and 1:1 diluted PhaZ containing spent supernatant (undetermined number of units) – already on day 1, the scaffolds were considerably enzymatically degraded. The longest degradation time was observed for the 1:100 dilution (the same as control scaffolds) and the 1:10 dilution where the scaffold damage was observed after 6 days. The obtained results indicated tunability of the degradation time by changing the enzyme concentration. For further experiments with cells, the 1:5 dilution with a 1 min incubation time was chosen as the degradation rate that provides the cells with enough time to attach to the fibers and start proliferating, before the scaffolds break down.

### 2.5 Cell Culture on Scaffolds

Before cell culture experiments, the swelling behavior of the PHA scaffolds was investigated to ensure that no severe liquid uptake, and therefore scaffold structure alterations, would occur during in vitro studies. None of the scaffold types revealed any major visible or weight‐based swelling (Figure S10 and Table S2, Supporting Information). Cell culture experiments were conducted on scaffolds printed with PHBV, PHBV+P34HB, and PCL as a control. Two designs, namely random and square mesh (Figure 1), were assessed during the cell culture studies. PCL was chosen as a comparison printing material as it has been widely used for cell culture studies, proving its biocompatibility and cell guidance properties when printed with MEW. The printing parameters used for the PCL scaffold controls are provided in Table S3 (Supporting Information).^[^ ^55^, ^56^ ^]^ The square scaffolds made of PCL and both PHBV and PHBV+P34HB revealed no statistical significance regarding fiber diameter and inter‐fiber distances (Figure S11, Supporting Information). The random scaffolds were assessed visually due to the difficulty of quantitative assessment for randomly deposited fibers. No clear differences concerning fiber placement and diameter were observed between the materials (Figure S12, Supporting Information).

**Figure 5:**
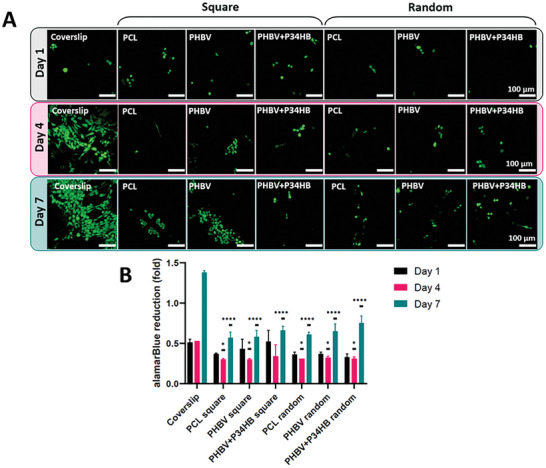
A) Fluorescence microscopy images of FDA staining conducted on live HDF cells grown on PCL, PHBV, and PHBV+P34HB scaffolds printed with two architectures, and glass coverslips (control). Scale bars: 100 µm; B) Cell metabolic activity assay. Data are presented as mean ± SD, *n* = 3. The statistically significant differences in graph B refer to a specific sample in comparison with the coverslip. Significant statistical difference: *p ≤ 0.05; ****p ≤ 0.0001.

The HDF cells were grown on the scaffolds for 7 days. Fluorescent staining of living cells with fluorescein diacetate (FDA) revealed living cells on all the scaffolds (**Figure** **5**A). On day 7 there were visibly more cells on PHBV and PHBV+P34HB in comparison to PCL. It was observed that the number of cells on the glass coverslip controls was higher than on the scaffolds. However, the coverslips are not permeable (uniform surface), whereas the scaffolds had relatively large gaps between the fibers, leading to low efficiency of cell attachment in the latter case. Cell metabolic activity was investigated via the alamarBlue assay on days 1, 4, and 7 (Figure 5B). The activity visibly increased over time for all the samples. However, the alamarBlue reduction on day 4 and day 7 was lower for HDF cells grown on the scaffolds in comparison to the flat coverslips. This result was attributed to the lower seeding efficiency on the printed scaffolds in comparison to the coverslips. Notably, there was no statistically significant difference between cells grown on PCL and PHA scaffolds.

**Figure 6:**
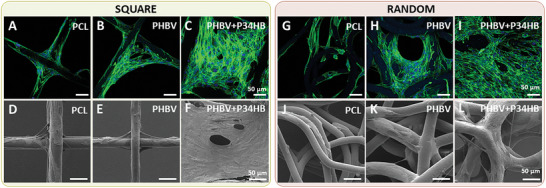
A–C,G–I) Immunofluorescence staining of HDF cells grown on PCL, PHBV, and PHBV+P34HB scaffolds with square and random designs on day 7. Dyes: DAPI (nucleus, blue), phalloidin (cytoskeleton, green). Scale bars: 50 µm. D–F,J–L) SEM images of HDF cells grown on PCL, PHBV, and PHBV+P34HB scaffolds with square and random designs on day 7. Scale bars: 50 µm.

Next, cell morphology was investigated using immunofluorescence staining (of nuclei and the cytoskeleton) and SEM (**Figure** **6**). On the square scaffolds, HDF cells grew from the corners of the squares toward the center of the fibers (Figure 6A–C). Similarly, in random scaffolds, the cells bridged the gaps between fibers placed close together first, before proceeding to form a continuous cell layer (Figure 6G–I). Moreover, it was observed that HDFs grown on PHBV+P34HB scaffolds more often stretched over the gaps of both square and random geometries (Figure 6C,F,I,L). Even though the cells were able to bridge the gaps better in the PHBV+P34HB samples than in the PHBV and PCL samples, their distribution was not homogeneous. This was likely caused by the low seeding efficiency of highly porous meshes, a commonly reported challenge of MEW scaffolds.^[^ ^57^ ^]^ The favorable cell bridging of the gaps between pores in PHBV+P34HB samples can be explained by the increased surface roughness of the fibers. It was reported in the literature that subjecting PCL scaffolds to sodium hydroxide can introduce texture to the otherwise smooth PCL surface, leading to improved cell attachment and growth.^[^ ^16^, ^58^ ^]^ The measured roughness was significantly higher for PHBV and PHBV+P34HB than for untreated PCL (Figure S13A,D,G, Supporting Information). These findings were further supported by surface profile analyses (Figure S13B,E,H, Supporting Information) and profile graphs (Figure S13C,F,I, Supporting Information), where the PCL plots remained uniform with minimal variation as shown by the gray value range of 50 units, whereas the ranges for PHBV and PHBV+P34HB were 100 and 150 units, respectively.

**Figure 7:**
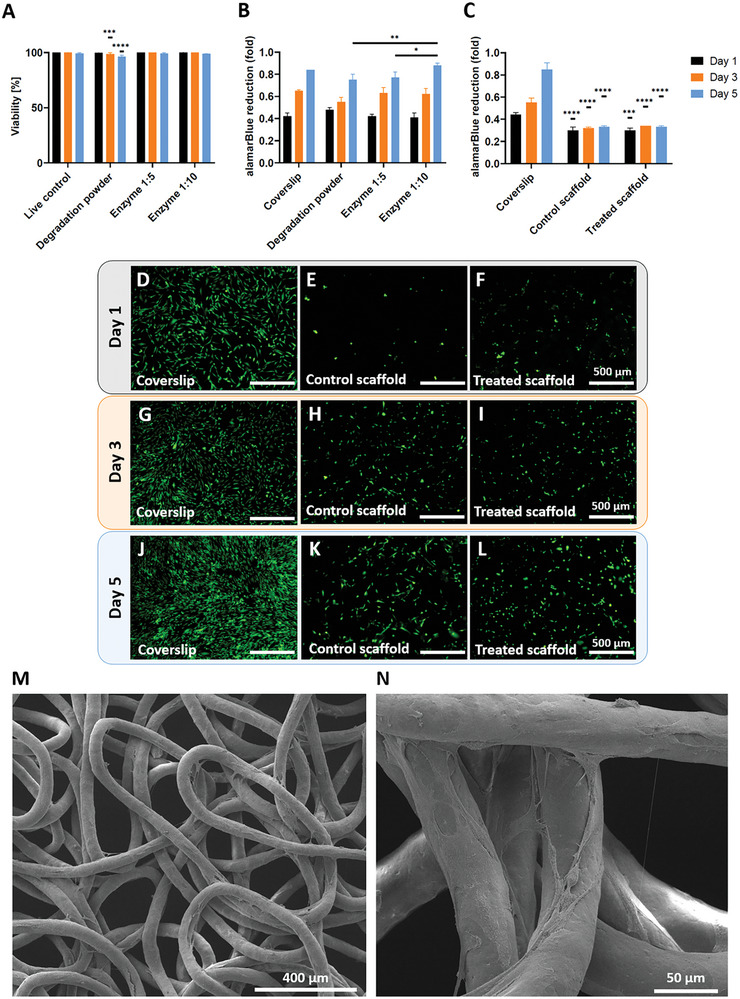
A) Cell viability of HDF cells performed via Live/Dead assays, where the degradation powder group on days 3 and 5 is significantly different than all other groups; B) Cell metabolic activity assay conducted on HDF cells exposed to the enzymatic degradation powder and enzyme solution diluted with medium into 1:5 and 1:10 concentration compared to control. Data are presented as mean ± SD, *n* = 6 (or *n* = 4 for dead control on day 1; viability) and *n* = 3 (metabolic activity). Significant statistical difference: *p ≤ 0.05; **p ≤ 0.01; ***p ≤ 0.001; ****p ≤ 0.0001; C) Cell metabolic activity assay of FDA staining conducted on live HDF cells grown on a PHBV+P34HB scaffold, previously subjected to enzymatic degradation in 1:5 enzyme‐to‐medium solution, compared to controls grown on PHBV+P34HB and controls on coverslips. The statistically significant differences refer to a specific sample in comparison with the coverslip controls; and D‐L) Fluorescence microscopy images of FDA staining. Scale bars: 500 µm; M,N) SEM images of HDF cells grown on PHBV+P34HB scaffolds treated with enzymes, after 5 days of culture; Data are presented as mean ± SD, *n* = 3. Significant statistical difference: ***p ≤ 0.001; ****p ≤ 0.0001.

The objective of the study is to develop scaffolds for biodegradable implants. Accordingly, the residual enzymatic degradation powder and PHA‐degrading enzymes were examined in combination with cells to assess the biocompatibility of the system. To assess the biocompatibility of the degradation products, the residue powder from enzymatic degradation collected from PHBV+P34HB scaffolds (1 mg mL^−1^ concentration) or an enzyme solution was added to HDF cells cultured for two days. The cells displayed high viability (≈100%) for all conditions (**Figure** **7**A). The metabolic activity measurements revealed comparable values in all conditions and an increase in cell activity over time (Figure 7B). These results indicate the absence of adverse effects from the enzyme and degradation products.

To investigate whether the use of enzymes on the PHA scaffolds influences cellular behavior, PHBV+P34HB random scaffolds were incubated in an enzyme solution diluted with the complete medium in a 1:5 ratio for 1 min, prior to HDF cell seeding. The cells were cultured on scaffolds for 5 days and investigated via FDA staining and metabolic activity assay. The FDA‐stained cells were found on both scaffold types with no apparent differences between treated and untreated ones (Figure 7C–K). The number of labeled cells increased between each time point. Analysis of metabolic activity showed that enzymatically treated scaffolds displayed significantly less alamarBlue reduction compared to coverslip controls (Figure 7L), which can be related to lower seeding efficiency on the scaffolds. However, no significant differences were observed between treated scaffolds and control scaffolds, which suggests that enzyme treatment did not have any negative influence on the metabolic activity of the HDF cells over the course of 5 days. Furthermore, the morphology of HDF cells on enzymatically treated PHBV+P34HB scaffolds was analyzed using SEM imaging, as shown in Figure 7M,N. After 5 days of culture, the cells populated the scaffold, uniformly covering the fibers (Figure 7M). The cells displayed an elongated and flattened morphology, adhering to single fibers and extending across the inter‐fiber gaps (Figure 7N). Similar cell morphology was observed after the 5‐day culture on the untreated PHBV+P34HB scaffold with a random design (Figure 6L). These results indicate that the enzymatic treatment did not affect cell morphology.

Similarly, the influence of enzyme addition (1:5 dilution in medium, 1‐min incubation) to the PHBV+P34HB random scaffolds, combined with HDF cell culture, was investigated over an 8‐day period (Figure S14, Supporting Information). The metabolic activity of HDF cells on treated scaffolds was compared to control scaffolds and cells cultured on glass coverslips. For all sample types, metabolic activity increased significantly over time, indicating cell proliferation (Figure S14A, Supporting Information). Notably, no significant differences were observed among the three groups throughout the 8 days, suggesting that the addition of the active enzyme does not negatively impact cell activity. Representative fluorescence staining images of HDF cells cultured for 8 days on control (Figure S14B, Supporting Information) and treated scaffolds (Figure S14C, Supporting Information) demonstrate similar cell coverage of the fibers, with no visible changes in cellular behavior. SEM imaging of enzymatically treated scaffolds revealed signs of degradation, such as cavities and fiber breakage after 8 days (Figure S14, Supporting Information, yellow arrows), which were absent in control conditions and in previous cell studies without the enzyme addition (Figure 6L). These observations align with the fiber breakage reported in Figure 4D, where most of the scaffold structure remained intact, but localized, inhomogeneous damage was visible. However, no significant surface roughness changes were noted in the treated samples, as shown in Figure S15 (Supporting Information). The gray values observed for both sample types varied within a similar range of 150 [‐], likely due to the enzyme dilution limiting the number of occupied binding sites on the fibers. This contrasts with the results in Figure 3B, where filaments were exposed to undiluted enzyme solution for a longer period, allowing more enzyme adherence and hydrolysis sites, resulting in clear surface structure modification and a visible increase in roughness. Furthermore, due to batch‐to‐batch enzyme variability or the presence of cells, the second stage of degradation (Figure 4D) in the 1:5 enzyme dilution occurred after 8 days, rather than 5. Interestingly, HDF cells attached to the areas of clear degradation (Figure S14E, Supporting Information, middle arrow), indicating that enzyme presence does not hinder cell attachment or growth.

Even though no immediate cytotoxic effects of the enzymatic degradation powder were found over 5 days, possible long‐term effects on cells in vivo cannot be excluded, as these have previously been reported in the literature.^[^ ^59^, ^60^, ^61^, ^62^, ^63^ ^]^ It has been reported that depending on shape, size, and concentration, BN shows toxicity. Hexagonal BN nanoparticles induced mild cytotoxicity on human normal skin fibroblast and Madin‐Darby Canine Kidney cells over 48 h at the concentrations of 0.2–0.4 mg mL^−1^.^[^ ^59^ ^]^ In the form of nanotubes, BN stimulated macrophages to generate numerous multinucleated giant cells, while fibroblasts exhibited elevated eosinophil levels.^[^ ^60^ ^]^ It was also shown that the cytotoxicity was elevated for the nanotubes with straight shapes enabling that enabled easy cellular uptake. Furthermore, a study by Mandarino et al. (2019) demonstrated that talc, another additive, increased reactive oxygen species production and altered the expression of macrophage genes related to cancer development.^[^ ^61^ ^]^ Similarly, TiO_2_ in the form of nanoparticles was deemed cytotoxic, as it induces the production of reactive oxygen species, which increased stress levels and resulted in genotoxicity.^[^ ^62^, ^63^ ^]^

Given the mechanical and thermal properties of the printed PHAs, a significant challenge arises in maintaining homogeneous and stable scaffolds throughout the printing process, due to the material's rapid degradation. This issue may restrict the size of printed scaffolds that can be accurately fabricated via the proposed setup. An increase in stiffness and brittleness of the scaffolds with printing time was observed, indicating that scaffolds printed at later time points will be more difficult to handle. This highlights again the importance of restricting the time PHAs are exposed to high temperatures. A possible approach to printing PHAs with less thermal degradation could be the use of MEW printers with local filament heating, instead of heating the bulk of the material.^[^ ^36^ ^]^ Such a solution would broaden the printability window. Moreover, it was reported that additional drying of the material before printing could be beneficial for restraining its degradation.^[^ ^64^ ^]^ Lastly, the availability of many PHA types (with over 150 identified PHA monomeric units) enhances the likelihood of finding compositions with higher temperature resistance. This makes them promising candidates for 3D printing and tissue engineering applications for biomedical uses, given their sustainability and biodegradability.^[^ ^4^ ^]^

In the future, more emphasis should be placed on synthesizing PHAs without any mineral additives and on performing cell studies on enzymatic degradation products over longer time periods, focusing on possible endocytosis of smaller non‐degraded PHA particles.

## 3 Conclusion

In this study, it is shown for the first time that PHAs, namely PHBV, and PHBV+P34HB, are well‐printable using MEW. Scaffolds with various architectures were successfully obtained from both materials. The lowest controlled inter‐fiber distance obtained for square scaffolds was 300 µm and ≈100 µm for randomly organized scaffolds. The fiber diameters ranged from ≈20 to 50 µm. Due to thermal degradation, the printing windows were between 20–90 min and 40–160 min for PHBV and PHBV+P34HB, respectively. PHBV+P34HB performed better as it exhibited a longer printability window and resulted in less brittle scaffolds. The Young's modulus of the printed scaffolds was dependent on the design and material used and ranged from 35 to 120 MPa for PHBV, and from 30 to 50 MPa for PHBV+P34HB. The brittleness of the samples was reduced by using scaffolds printed only at the beginning of the respective printing windows.

Scaffolds were successfully subjected to selective enzymatic degradation. To obtain a desirable degradation time, scaffold designs and enzyme‐to‐medium dilution ratios could be adjusted. The HDF seeded on the PHBV and PHBV+P34HB scaffolds showed improved metabolic activity comparable to gold‐standard PCL scaffolds throughout 7 days of culture. Moreover, the cell studies performed on PHBV+P34HB scaffolds subjected to enzymatic degradation prior to cell seeding revealed the biocompatibility of the proposed degradation process. These results suggest that pre‐programmed degradation of PHBV+P34HB scaffolds could be a feasible option for designing cell‐only (ex vivo) implants. Importantly, this study showed a novel approach to green chemistry by utilizing a bacteria‐produced polymer that can be printed into scaffolds via MEW, without using additional solvents and minimizing waste products. The obtained scaffolds are suitable substrates for cell culture and can be readily enzymatically degraded by PHA depolymerase. However, the importance of synthesizing PHAs without any undesirable additives when considering future biomedical applications is stressed. In addition, the fact that the development of good manufacturing practices for depolymerase production is essential when considering future in vivo studies is acknowledged. As the enzymatic degradation process leaves the cells intact, the materials can be used in the future for the production of ex vivo implants with patients' own stem cells and supporting scaffolds degrading on demand. Additionally, as bacterial production of the material eases the customization; different, optimized PHAs with more desirable mechanical and thermal properties, can be obtained. Prospectively, we envision that PHAs may serve as advanced substrates for skin grafts or as sacrificial materials for the fabrication of vascular channels. The latter holds significant promise for advancing research in the field of vascularization.

## 4 Experimental Section

### Scaffold Fabrication

All scaffolds were printed with a MEW printer (MELT A‐1204‐0001‐01D, Spraybase, Ireland). The parameters used for printing each material can be found in the Table S1 (Supporting Information). PHBV was purchased from PHAradox under the commercial name Enmat Y1000p. PHBV+P34HB was purchased from ColorFabb under the commercial name colorFabb allPHA/colorFabb PHA natural. The PHAs were stored in confinement together with silica gel to prevent water absorption and degradation, as it decreases the materials printability. Moreover, before printing, the PHAs in the heating chamber were exposed to a nitrogen purge.

### Tensile Testing

Six samples of each design (random, semi‐random, square) from both PHBV and PHBV+P34HB were used for the measurements. Tensile testing was performed on an Instron 68SC‐1 with the use of a 50 N load cell and with a crosshead speed of 5 mm min^−1^. The data was collected and processed using the Bluehill Universal software. The same settings were used for the investigation of square mesh designs printed with PHBV and PHBV+P34HB. Each scaffold was printed with 300 µm of inter‐fiber distance and two layers. For analysis, three replicates of each scaffold were used.

### Melt Rheology

An HR‐2 rheometer (TA Instruments, United States) equipped with a convection oven running with nitrogen was used to perform the melt rheological measurements. For all experiments, a stainless‐steel plate‐plate geometry with a diameter of 25 mm was used. Neat samples of PHBV, PHBV+P34HB, and PCL were loaded on the rheometer and heated to their corresponding printing temperature (180 °C for PHBV and PHBV+P34HB, and 100 °C for PCL) for ≈10 min until fully melted. Then, the gap was adjusted to have an adequate loading under the geometry. The PHBV, PHBV+P34HB, and PCL were heated for another 10, 30, and 10 min, respectively. A time sweep measurement was performed at a low deformation regime (1% strain and 1 rad s^−1^ frequency) and elastic modulus (G′), viscous modulus (G″), and complex viscosity were recorded to investigate the rheological changes of samples over time.

### Thermal Degradation of PHAs

Both PHBV and P34HB were thermally degraded by heating in an oven at 180 °C for 5 h to analyze the properties of the thermally degraded polymers.

### Fourier‐Transform Infrared (FTIR) Spectroscopy

Measurements were performed using a Bruker Platinum ATR VERTEX 70 FTIR Spectrometer. Spectra were taken from 400 to 4000 cm^−1^ with a resolution of 2 cm^−1^ and 64 scans. The spectra were processed in Excel, Microsoft Office 2019, version 1808. The FTIR spectra were transposed to absorbance and the data for the neat samples were matched to the main peak at 1720 cm^−1^ (assigned to C═O stretching) of the thermally degraded samples to show relative intensities.

### Thermal Gravimetric Analysis (TGA)

TGA5500 thermogravimetric analyzer from TA Instruments Inc., serial number 5500‐0057 was used for the measurements, which were performed under air conditions and either from 20 to 500 °C with a heating rate of 10 °C min^−1^ or at a constant temperature of 180 °C for 375 min.

### Differential Scanning Calorimetry (DSC)

Measurements were performed on an Instrument DSC Q1000 V9.8 Build 296, TA Instruments. Temperature scans were taken with a heating rate of 10 °C min^−1^. For each sample, three cycles were measured, from which the two latter cycles were shown to display the thermal behavior of the sample without thermal history. The data was processed using Universal Analysis software from TA Instruments. The melting temperature (*T_m_*), enthalpy of melting (Δ*H_m_*), crystallization temperature (*T_c_*), and enthalpy of crystallization (Δ*H_c_*) were determined from the endothermic and exothermic peaks.

### Gel Permeation Chromatography (GPC)

Neat and thermally degraded samples of PHBV and PHBV+P34HB were dissolved in chloroform by heating at 100 °C for 1 h in a Monowave 50 Synthesis reactor (Anton Paar, Germany), in thick‐wall capped glass vials supplied with the reactor. The final concentration of the samples was ≈30 mg mL^−1^. Polymer solutions were slightly turbid after heating and were filtered through 0.2 µm disc filters (Chromafil). Analysis was done by injecting 20 µL of the filtered polymer solution in an Agilent setup for GPC analysis (1100 series HPLC pump and autosampler, 1260 Infinity II RI‐detector). The eluent was chloroform (Sigma Aldrich, Chloroform for HPLC) at a flow rate of 1 mL min^−1^. Three SDV‐columns (300×8 mm) with pore sizes of 1000 Å, 100000 Å, and 10000000 Å (PSS, Germany) were connected in series and kept at 35 °C in a column oven (Shimadzu CTO‐10AC). A calibration curve was obtained using 12 narrow‐band polystyrene standards in the range 764–2460000 Da (PSS, Germany) that were run in duplicate. Using the Open Lab software (Agilent) a 3rd‐order polynomial calibration curve was created (*R* ^2^ > 0.9999) and the sample M_w_, M_n,_ and Đ results were calculated. A calibration control sample (M_w_ = 34000) was run twice and deviated less than 8% from the control value.

### Scanning Electron Microscopy – Energy‐Dispersive X‐Ray (SEM‐EDX)

Images were taken with FEI Quanta 400 FEG equipped with an EDAX Genesis V 6.04 X‐ray spectrometer. An accelerating voltage of 3, 5, or 10 (20) kV, low‐vacuum (Large Field Detector, 100 Pa water vapor), or high‐vacuum (Everhart‐Thornley Detector) was used after gold sputtering with a JEOL JFC‐1300 Auto Sputter Coater (30 mA, 45 s). The EDX analysis was performed with the same equipment as for SEM. For some of the EDX measurements in low‐vacuum mode, an X‐ray cone was used to enhance the local sensitivity of the chemical analysis.

Cellular samples for SEM imaging were collected on days 1, 4, and 7, washed with Dulbecco's phosphate‐buffered saline (DPBS) (Sigma–Aldrich, D8537, Netherlands), fixed with 2.5% glutaraldehyde in cacodylate buffer (ThermoFisher Scientific, 50‐259‐41) solution and kept in it until SEM preparation. This was done by dehydration using a row of ethanol/water mixtures with increasing ethanol ratio until 100% ethanol followed by drying using hexamethyldisilazane (HMDS) added first 1:1 to the samples in ethanol before transfer to 100% HMDS and a slow evaporation of HMDS under atmospheric conditions. The dried samples were fixed on an Al sample holder with double‐sided carbon tape before SEM imaging.

### Enzymatic Degradation

PHA depolymerase was produced using a recombinant *Bacillus subtilis* 168 strain. The *phaZ* gene from *Bacillus* sp. NRRL B‐14911, encoding a secreted PHA depolymerase (PhaZ), was genomically integrated using plasmid pDR111 (D. Rudner, Harvard University Boston).^[^ ^65^ ^]^ Gene expression was under the control of the IPTG‐inducible hyperspank promoter. The strain was cultured in lysogeny broth (LB) consisting of 1% (w/v) peptone (VWR), 0.5% (w/v) yeast extract (VWR), and 1% (w/v) sodium chloride (Boom) dissolved in demineralized water. The LB was supplemented with 100 µg mL^−1^ spectinomycin.

Liquid culture of *B. subtilis* 168: *phaZ* was induced at an OD600 of 0.4 with 1 mM IPTG for 16 h and subsequently centrifuged at 3320 x *g* (Eppendorf 5810R) for 20 min. The spent supernatant was supplemented with 0.5 gr mL^−1^ (NH_4_)_2_SO_4_ to precipitate proteins. The pellet was resuspended in 0.5 volume of fresh LB supplemented with 50 µg mL^−1^ kanamycin and used for impregnating MEW meshes.

PHBV or PHBV+P34HB was sterilized in 70% ethanol and air dried, before submerging in filtered supernatant (0.22 µm filter) of the PHA depolymerase, either with or without DMEM (Gibco, 31966, Netherlands), for a specific amount of time at 37 °C in a humidified atmosphere of 5% CO_2_. Next, the PHBV or PHBV+P34HB was washed with either DMEM (Gibco, 31966, Netherlands) or DPBS (Gibco, 14190094, Netherlands) to remove any other remnants from the bacteria culture. PHBV or PHBV+P34HB was then placed in DMEM and kept at 37 °C. The solution was replenished every time a noticeable color change of the pH indicator occurred due to acidification.

### X‐Ray Diffraction (XRD)

For the analysis, a Bruker D8 ADVANCE in Bragg‐Brentano geometry, operating with a Cu Kα radiation source (λ = 1.54 Å) and a Lynxeye XE‐T detector was used. Since it was a small, it was propped on a heightened non‐detectable carbon‐based platform and not rotated along its horizontal axis to stay well aligned with the laser (which was normally done to avoid directional preferences of the crystal planes), so a clear measurement could be taken.

### Transmission Electron Microscopy – Energy‐Dispersive X‐Ray (TEM‐EDX)

For TEM, a droplet of the aqueous dispersion of the degradation residue was placed on a holey carbon‐supported TEM grid (Plano, S147‐4, Germany) and dried under ambient conditions. Bright‐field TEM images and EDX point analyses were performed using a JEOL JEM‐2100 LaB6 microscope equipped with a Thermo Noran NSS7 X‐ray spectrometer and a Gatan Orius SC1000 CCD camera at 200 kV accelerating voltage.

### Cell Culture

Cell culture was performed with the use of primary adult HDF derived from a 27‐year‐old Caucasian female (Innoprot, P10858, Spain) cultured according to the protocol from the provider. Shortly, the cells were cultured in a T75 flask coated with 2 µg cm^−2^ poly‐L‐lysine with DMEM (Gibco, 31966, Netherlands) supplemented with 10% fetal bovine serum (Sigma‐Aldrich, F9665, Netherlands), 1% penicillin−streptomycin (10000 U mL^−1^, Gibco, 15140148, Netherlands), and 1 ng mL^−1^ fibroblast growth factors (Gibco, PHG0367, Netherlands). Culture medium which was refreshed every 2–3 days. The cells were kept at 37 °C in a humidified atmosphere of 5% CO_2_, until over 90% confluence was reached.

### Biocompatibility

For biocompatibility studies, two types of scaffolds (square and random architectures) were melt electrowritten with PHBV, PHBV+P34HB, and PCL. Each scaffold was cut into smaller 0.5×1 cm rectangular pieces, which were then placed inside of a suspension 24‐well plate. As controls, glass coverslips (Epredia, CB00120RA120MNZ0, Netherlands) were used. Scaffolds and coverslips were sterilized with 70% ethanol for 15 min. Each sample was then washed three times with DPBS, and once with the cell culture medium. The HDF cells at passage 4 were seeded on scaffolds at a density of 48000 cells cm^−2^, and on top of the coverslips at a density of 24000 cells cm^−2^. The cells were grown for periods of 1, 4, and 7 days, followed by cytotoxicity, and metabolic activity assays at each time point.

The studies of HDF cells subjected to enzymatic degradation powder residues (1 mg mL^−1^) and enzymes (1:5 and 1:10 dilutions in DMEM) were as follows: Glass coverslips were sterilized with 70% ethanol for 15 min and washed three times with DPBS. The HDF cells at passage 6 were seeded on the coverslips at a density of 21000 cells cm^−2^. After 48 h the cells were exposed to enzymatic degradation powder (3 h incubation) or enzymes (1 min incubation followed by three DMEM washes). The cells were kept in culture for 5 days.

The studies of HDF cells grown on scaffolds incubated in enzyme solution were as follows: The PHBV+P34HB random scaffolds were cut into smaller 0.5 × 1 cm rectangular scaffolds, which were then placed inside a suspension 24‐well plate, together with control coverslips and sterilized with 70% ethanol for 15 min, followed by three DPBS washes. The scaffolds were incubated in an enzyme solution (1:5 dilution with DMEM) for 1 min and washed with complete DMEM prior to cell seeding. The cells at passage 6 were seeded on scaffolds at a density of 48000 cells cm^−2^, and on top of the coverslips at a density of 24000 cells cm^−2^.

The cytotoxicity was investigated via the Live/Dead assay. Briefly, a solution of FDA (ThermoFisher Scientific, F1303, Netherlands) was prepared by dissolving it in acetone to a concentration of 5 mg mL^−1^, followed by the addition of DPBS to obtain a final concentration of 20 µg mL^−1^. A solution of propidium iodide (PI) (ThermoFisher Scientific, P1304MP, Netherlands) was prepared by dissolving it in PBS to a concentration of 20 µg mL^−1^. The cells were incubated in the combination of both solutions, 0.2 mL of prepared FDA solution added to 0.06 mL of PI solution in 24‐well plates and 0.1 mL of prepared FDA solution added to 0.03 mL of PI solution in 48‐well plates, for 10 min. Next, the samples were washed twice with DPBS and imaged via a confocal microscope with 20x magnification (Zeiss 710 Laser Scanning Microscope, Germany). As controls, cells grown on coverslips (live control) and cells grown on coverslips treated with 70% ethanol for 10 min (dead control) were used. Six images per sample were used for analysis. Images were analyzed by Fiji software via functions “watershed” and “analyze particles”. Cell viability was calculated with the use of the following equation: *% viability = [(number of live cells)/(total number of cells)] × 100*. For visualization of FDA staining only the green channel was chosen.

Metabolic activity was investigated via the alamarBlue assay. The alamarBlue reagent (ThermoFisher Scientific, DAL1025, Netherlands) was added in a 1:10 ratio to the cell culture medium and incubated at 37 °C for 4 h. After the incubation, the medium from each well was transferred to another well plate and the absorbance at 570 nm, using 600 nm as the reference wavelength, was measured with a microplate reader (Tecan Spark M10 or BioTek Synergy H1 multimode microplate reader with Gen5 3.08 software).

### Immunofluorescence

Immunofluorescence of the samples on days 1 and 7 was investigated in the following manner. First, the samples were fixed with a 10% (w/v) formalin solution (HT501128, Sigma–Aldrich, Netherlands) for 15 min, after being washed with DPBS. After incubation, the formalin solution was removed, and the samples were washed twice with DPBS. Subsequently, the samples were incubated in 0.1% Triton 100X (v/v) (X100‐5ML, Sigma–Aldrich, 9002‐93‐1, Netherlands) in DPBS for 15 min, followed by three DPBS washes. Then 3% BSA (Sigma‐Aldrich, A9418, Netherlands) in DPBS was used to incubate the cells for 15 min, followed by three more DPBS washes. Next, the samples were incubated with AlexaFluor 488 phalloidin (Cell Signaling Technology, 8878S, USA) solution in DPBS (1:100 dilution) for 90 min. Then the samples were washed three times with DPBS and incubated with DAPI (Abcam, ab228549, Netherlands) solution in DPBS (1:1000 dilution) for 10 min. Lastly, the samples were washed three times with DPBS and kept in DPBS at 4 °C until imaging. The samples were imaged with a confocal microscope (Zeiss 710 Laser Scanning Microscope, Germany). For each sample, a z‐stack of 30 slices with 40x magnification was taken with the use of the same set of settings for each of the four dyes. Those 30 stacks were used to create composites, apart from the PHBV+P34HB sample with a square mesh, for which only stacks 11–14 were used.

### Surface Roughness

The surface roughness of printed fibers was analyzed through Fiji software, based on SEM images. The representative surface plots were generated with the “Surface Plotter” command and the profile graphs were generated through the “Plot profile” command.

### Statistical Analysis

For the fiber diameter and inter‐fiber distance measurements four SEM images were chosen (apart from PHBV+P34HB square and semi‐random scaffolds, from which two images were chosen), from which 20 random measurements were taken to derive mean values ± SD. Six values from tensile tests for each sample, except for semi‐random PHBV scaffolds, were used to derive the mean Young's modulus value and its standard deviation. The results from the same geometries but from two polymers were then analyzed via one‐way ANOVA with Tukey's post hoc test. For the comparison of square PHBV and PHBV+P34HB scaffolds printed at different time points, three replicates were used and analyzed via one‐way ANOVA with Tukey's post hoc test. The results from viability and metabolic activity assays were analyzed via two‐way ANOVA with Tukey's post hoc test.

## Conflict of Interest

The authors declare no conflict of interest.

## Supporting information



Supporting Information

## Data Availability

The data that support the findings of this study are available from the corresponding author upon reasonable request.
