# Supplementary material for: Melt Electrowriting of Polyhydroxyalkanoates for Enzymatically Degradable Scaffolds
Source: Adv Healthc Mater. 2024 Nov 12;14(6):2401504. doi: 10.1002/adhm.202401504 (PMC11874678; doi:10.1002/adhm.202401504)
Supplement: Supplementary file 1 — Supporting Information [file ADHM-14-0-s001.pdf]

ADVANCED HEALTHCARE MATERIALS

Supporting Information

for *Adv. Healthcare Mater.*, DOI 10.1002/adhm.202401504

Melt Electrowriting of Polyhydroxyalkanoates for Enzymatically Degradable Scaffolds

Magdalena Z. Gładysz, Didi Ubels, Marcus Koch, Armin Amirsadeghi, Frederique Alleblas, Sander van Vliet, Marleen Kamperman, Jeroen Siebring, Anika Nagelkerke and Małgorzata K. Włodarczyk-Biegun**

Supporting Information

Melt Electrowriting of Polyhydroxyalkanoates for Enzymatically Degradable Scaffolds

Magdalena Z. Gładysz[1,2], Didi Ubels[1], Marcus Koch[3], Armin Amirsadeghi[1], Frederique Alleblas[2], Sander van Vliet[4], Marleen Kamperman[1], Jeroen Siebring[4]*, Anika Nagelkerke[2], Małgorzata K. Włodarczyk-Biegun[1,5]*

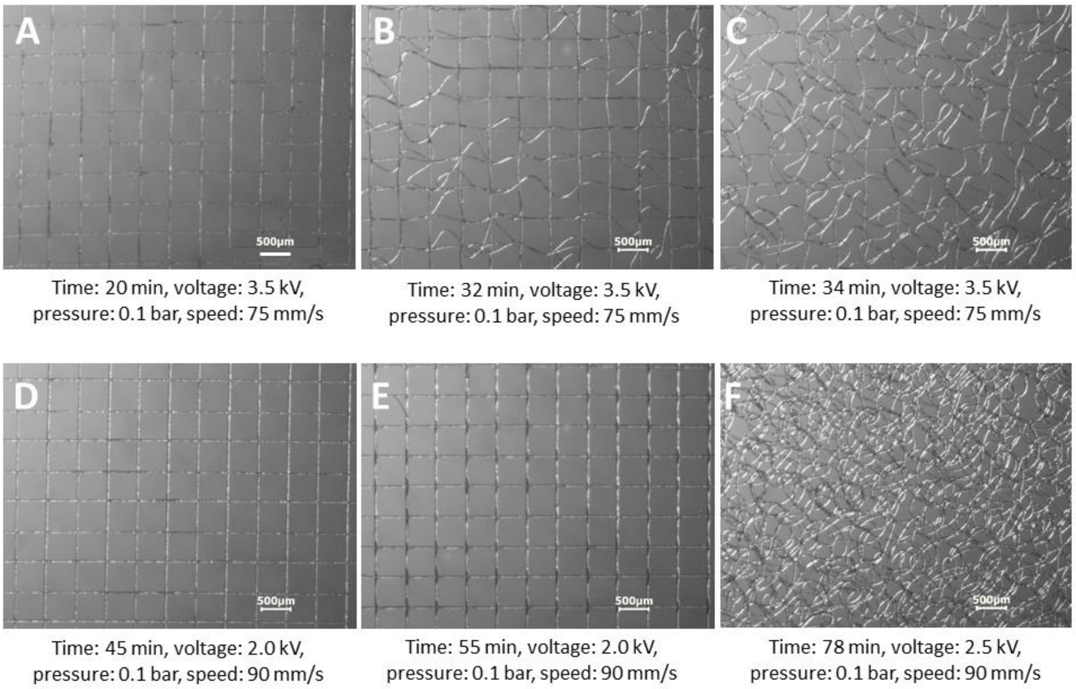

Figure S1. Brightfield microscopy images of melt electrowritten PHBV scaffolds printed with the same settings of temperature (180 °C), nozzle size (0.25 mm), nozzle-to-collector distance (4 mm), number of layers (4) and inter-fiber distance (500 µm). The changed parameters are shown below the images.

To correlate the thermal degradation of PHBV and PHBV+P34HB to their printing behavior, melt rheology was performed after relevant heating times. For PHBV, the polymer was pre-heated for 20 minutes at 180 °C and then a time sweep was performed at 180 °C for 80 minutes. For PHBV+P34HB, the polymer was pre-heated for 40 minutes at 180 °C and then a time sweep

was performed at 180 °C for 120 minutes. As a control experiment, PCL was pre-heated for 20 minutes at 100 °C and then a time sweep was performed at 100 °C for 120 minutes.

The results indicated that the complex viscosity of both PHBV and PHBV+P34HB decreased by approximately one order of magnitude during 80 and 120 min, respectively, which correspond to their respective printing time (Figure S2A, B). The complex viscosity of the PCL control remained constant over measurement time, showing material stability at the printing temperature (Figure S2C). These results are in agreement with the observation during printing and the GPC experiment. It is expected that a drop in molecular weight has a significant effect on the viscosity of polymer melts.[66] It was concluded that thermal degradation of PHBV and PHBV+P34HB through chain scission led to a more liquid-like melt behavior of the jet which could be distorted more easily under the electric field during MEW. These findings are also in agreement with previous studies of Daneshfar et. al. The authors showed that at constant MEW parameters using lower molecular weight PCL led to an unstable polymer jet (i.e. coiling) while a higher molecular weight resulted in stable jet formation.[67]

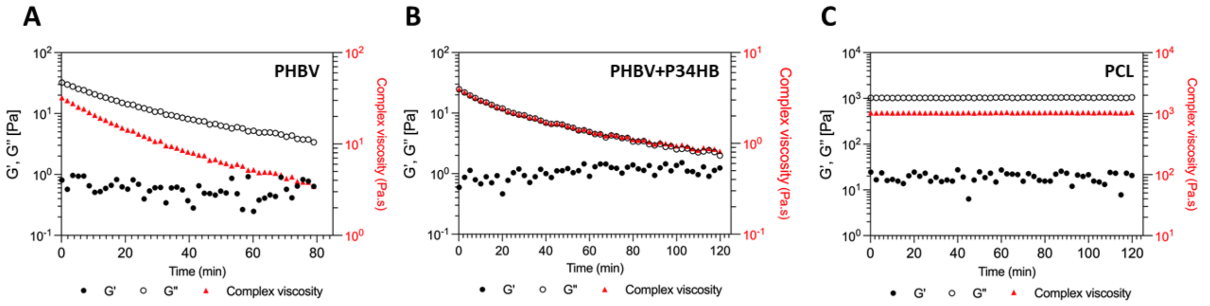

Figure S2. Time sweep rheology measurements for A) PHBV at 180 °C, B) PHBV+P34HB at 180 °C, and C) PCL at 100 °C under low deformation regime (1% strain and 1 rad/s). G' corresponds to the storage modulus and G'' corresponds to the loss modulus. Measurement times for PHBV and PHBV+P34HB corresponded to the printability windows established for these polymers.

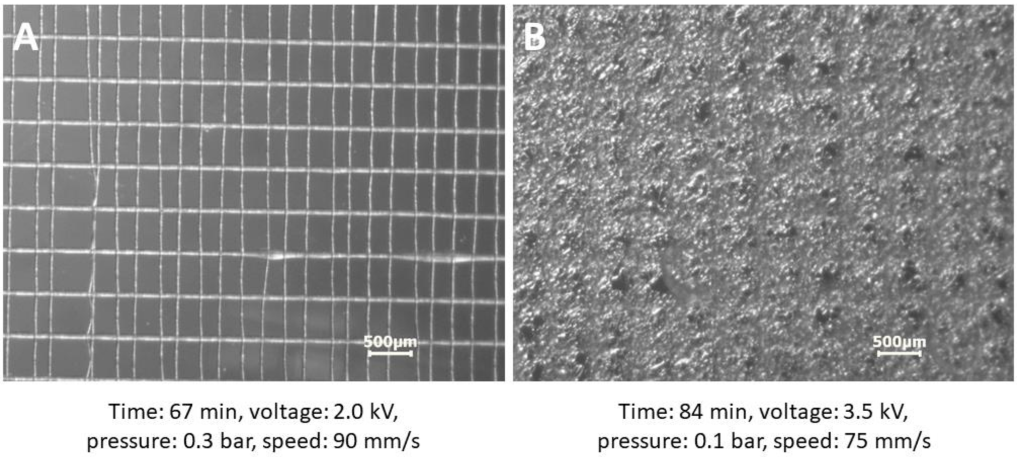

Figure S3. Brightfield microscopy images of melt electrowritten PHBV scaffolds printed after the heating time exceeded 60 minutes. Both scaffolds were printed with the same settings of temperature (180 °C), nozzle size (0.25 mm), nozzle-to-collector distance (4 mm), number of layers (4) and inter-fiber distance (300 µm). The flaws in the printed scaffolds can be seen A) in uneven fiber distribution and fluctuation in fiber diameters, and B) in fibers fused together.

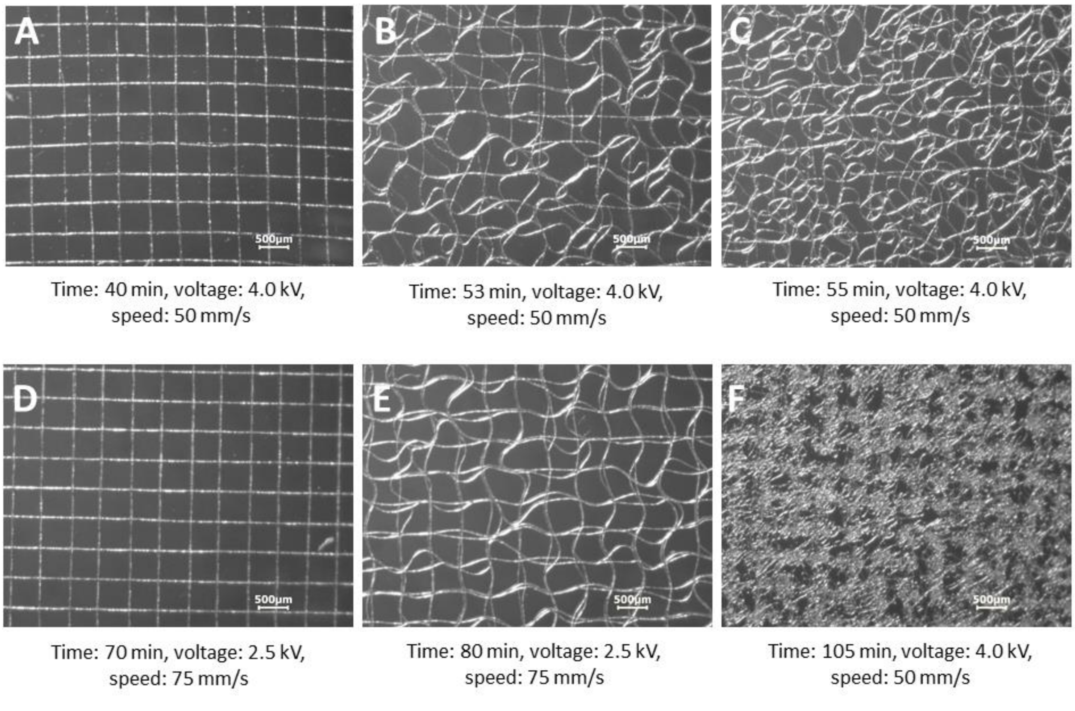

Figure S4. Brightfield microscopy images of melt electrowritten PHBV+P34HB scaffolds printed with the same settings of temperature (180 °C), nozzle size (0.25 mm), nozzle-to-collector distance (4 mm), number of layers (4), inter-fiber distance (500 µm) and pressure (0.14 bar). The changed parameters are listed in the figure descriptions.

Table S1. Printing parameters for the scaffolds shown in Figure 1.

Material	Random	Semi-random	Square
**PHBV**	Temperature: 180 °C<br>Voltage: 2.5 kV<br>Pressure: 0.1 bar<br>Speed: 90 mm s^{-1}<br>Nozzle size: 0.25 mm<br>Nozzle-collector distance: 4 mm<br>Inter-fiber distance: 500 µm<br>Layers: 4<br>Heating time point: 90 min	Temperature: 185 °C<br>Voltage: 3.5 kV<br>Pressure: 0.1 bar<br>Speed: 80 mm s^{-1}<br>Nozzle size: 0.25 mm<br>Nozzle-collector distance: 4 mm<br>Inter-fiber distance: 750 µm<br>Layers: 2<br>Heating time point: 75 min	Temperature: 180 °C<br>Voltage: 3.0 kV<br>Pressure: 0.1 bar<br>Speed: 75 mm s^{-1}<br>Nozzle size: 0.25 mm<br>Nozzle-collector distance: 4 mm<br>Inter-fiber distance: 300 µm<br>Layers: 4<br>Heating time point: 37 min
**PHBV+P34HB**	Temperature: 180 °C<br>Voltage: 2.5 kV<br>Pressure: 0.09 bar<br>Speed: 50 mm s^{-1}<br>Nozzle size: 0.25 mm<br>Nozzle-collector distance: 4 mm<br>Inter-fiber distance: 500 µm<br>Layers: 6<br>Heating time point: -	Temperature: 180 °C<br>Voltage: 4.0 kV<br>Pressure: 0.08 bar<br>Speed: 50 mm s^{-1}<br>Nozzle size: 0.25 mm<br>Nozzle-collector distance: 4 mm<br>Inter-fiber distance: 500 µm<br>Layers: 6<br>Heating time point: -	Temperature: 180 °C<br>Voltage: 4 kV<br>Pressure: 0.2 bar<br>Speed: 75 mm s^{-1}<br>Nozzle size: 0.25 mm<br>Nozzle-collector distance: 4 mm<br>Inter-fiber distance: 300 µm<br>Layers: 4<br>Heating time point: 58 min

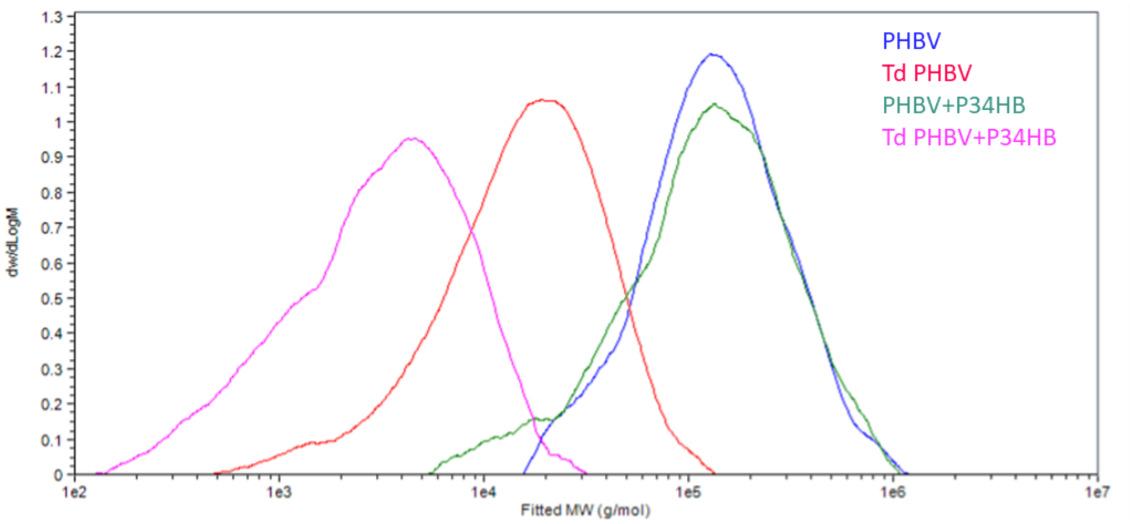

Figure S5. GPC molecular weight distribution plots. A clear shift to the left corresponding to lower molecular weight values for the thermally degraded (Td) samples was observed. The shift was more substantial for PHBV+P34HB compared to PHBV.

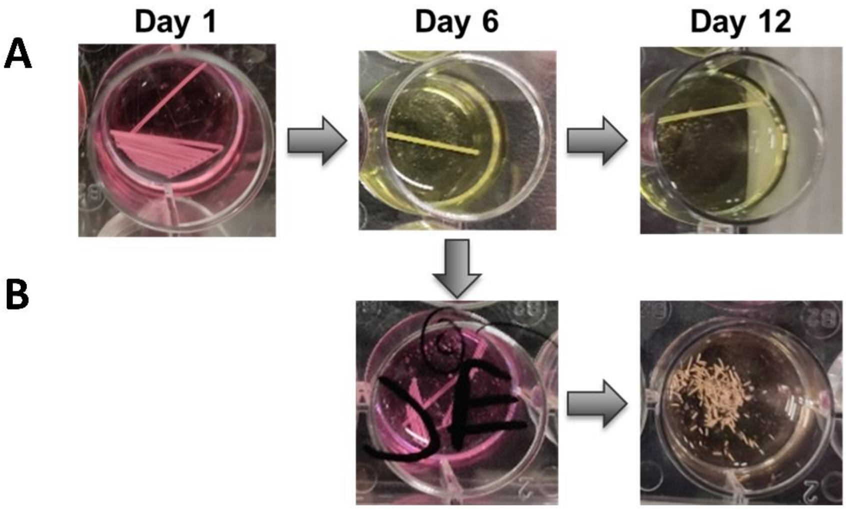

Figure S6. Images of PHBV+P34HB filaments in wells at various time points. PHBV+P34HB filaments, previously treated enzymatically, were incubated in medium in well A for 6 days. During this time, the medium changed from pink to yellow due to acidification. Some filaments were then transferred to a new well with fresh medium (well B), while others remained in well A. By day 12, the filaments in well B showed further degradation, while those in well A remained intact. This indicates that PhaZ activity is pH-dependent and can be restored with pH adjustment. It also suggests that enzyme activity persists on the chosen PHA (here PHBV+P34HB) following the initial incubation.

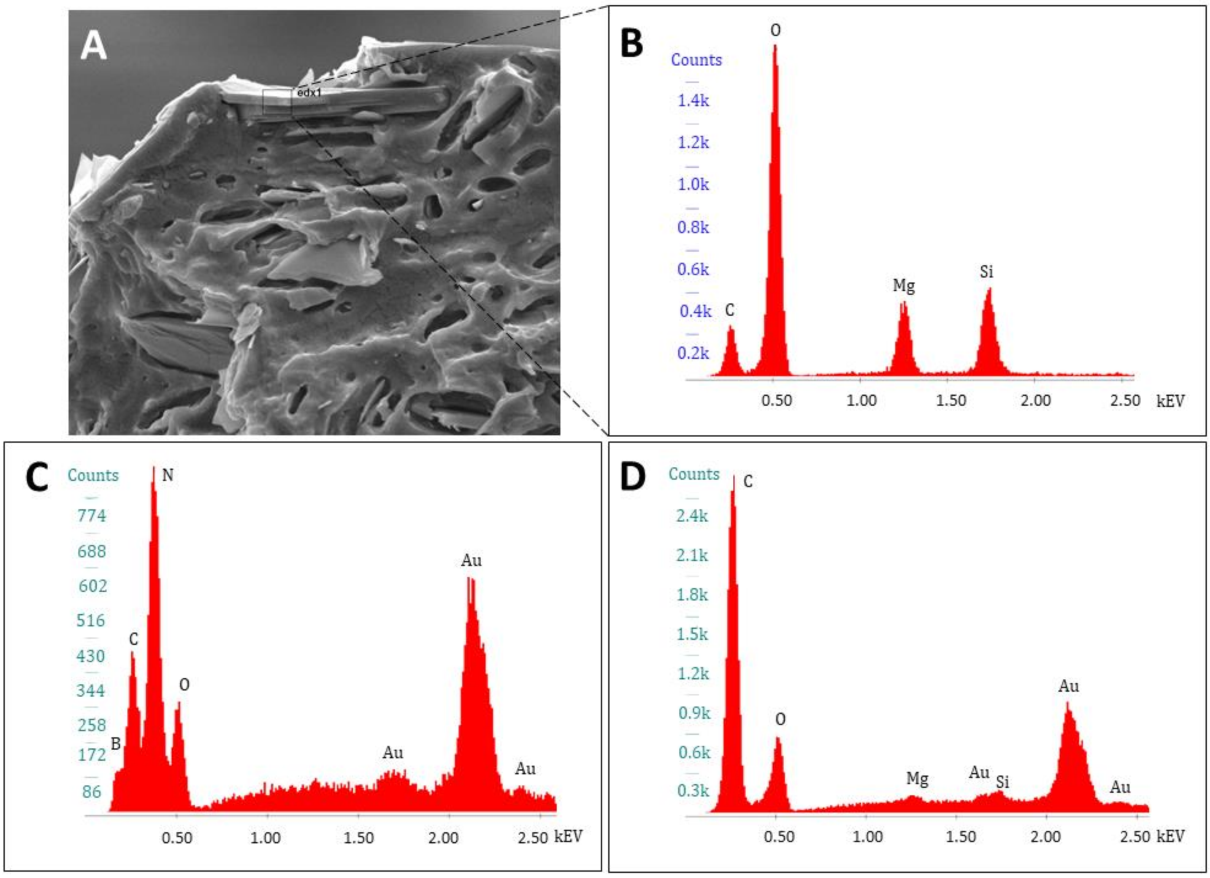

Figure S7. A) SEM image of PHBV+P34HB fiber cross-section, B) Corresponding EDX spectrum, C-D) EDX spectra of other PHBV+P34HB fibers.

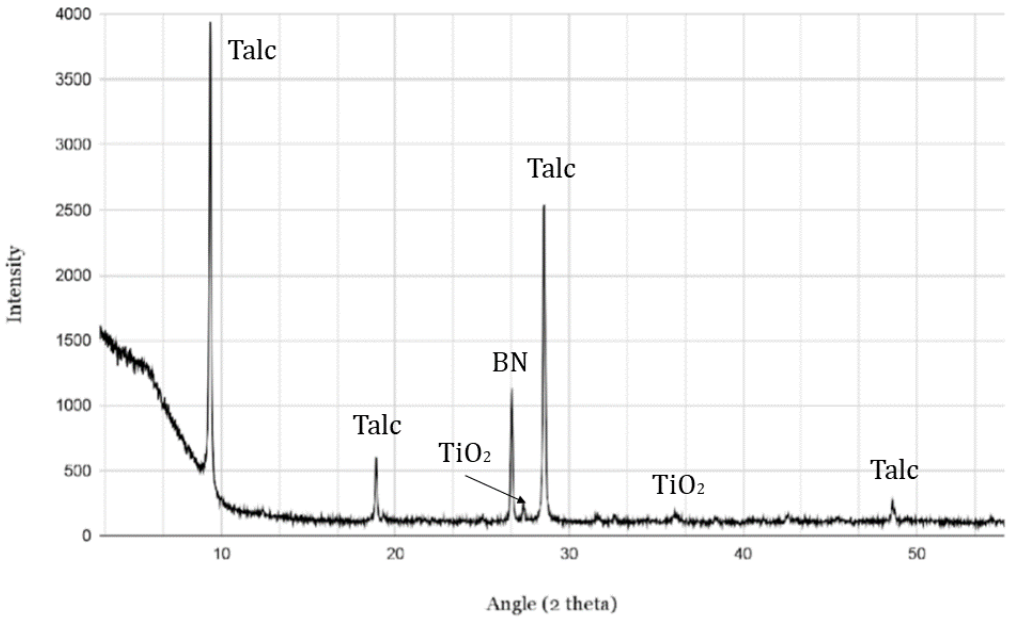

Figure S8. XRD of the enzymatic degradation powder presented with identified peaks.

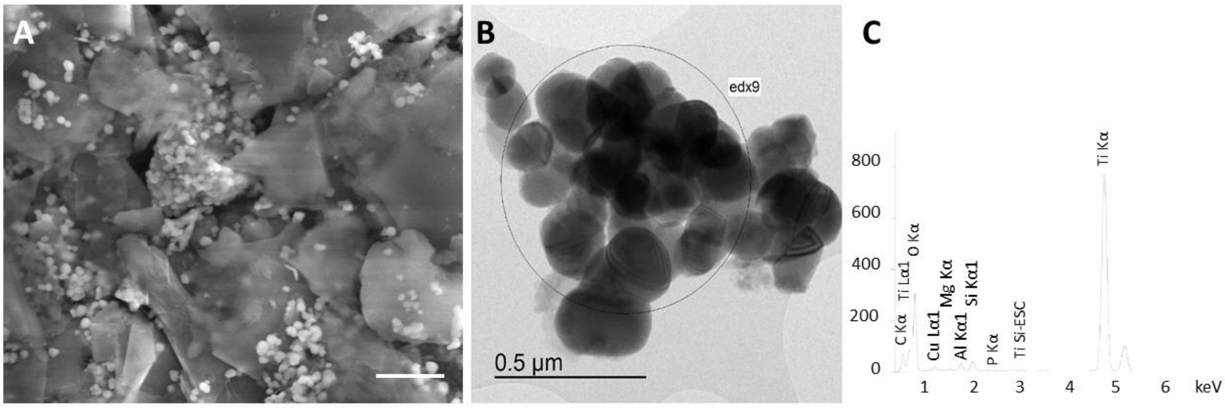

Figure S9. A) SEM image of degradation powder. Scale bar: 2 µm. B) TEM image coupled with C) EDX spectrum showing TiO_2 nanoparticles.

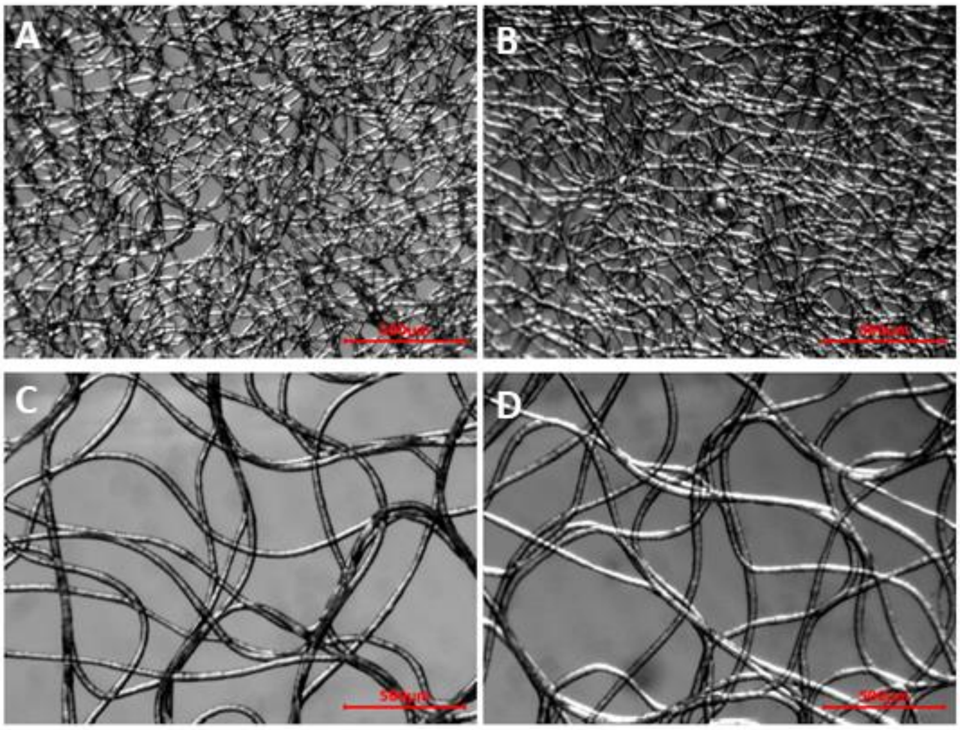

Figure S10. Brightfield microscopy images of PHA scaffolds after being submerged in DPBS or DMEM (Gibco, 31966, Netherlands) for 72 hours to investigate their swelling properties. A) PHBV scaffold before and (B) after being submerged in DPBS; C) PHBV+P34HB scaffold before and D) after being submerged in DMEM.

Table S2. Weight-based swelling results for PHA scaffolds after being submerged in DMEM or DPBS (Gibco, 31966, Netherlands) for 72 hours. Data are presented as the average weight percentage decrease or increase ± SD of PHBV (n = 1) and PHBV+P34HB (n = 3). The slight decrease in weight for PHBV was attributed to its brittleness, making part of the scaffolds break

off during handling. The slight increase in weight of PHBV+P34HB was attributed to a minor uptake of liquid.

Medium	PHBV	PHBV+P34HB
DMEM	Decrease of 0.9 wt%	Increase of 2.8 ± 2.1 wt%
DPBS	Decrease of 6.1 wt%	Increase of 5.3 ± 2.2 wt%

Table S3. Printing parameters for the PCL scaffold controls.

Parameter	Random design	Square design
Temperature	100 °C	100 °C
Voltage	7.0 kV	5.85 kV
Pressure	0.2 bar	0.3 bar
Speed	3 mm s^{-1}	10 mm s^{-1}
Nozzle size	0.30 mm	0.30 mm
Nozzle-collector distance	5 mm	4 mm
Inter-fiber distance	200 µm	300 µm
Layers	4	4

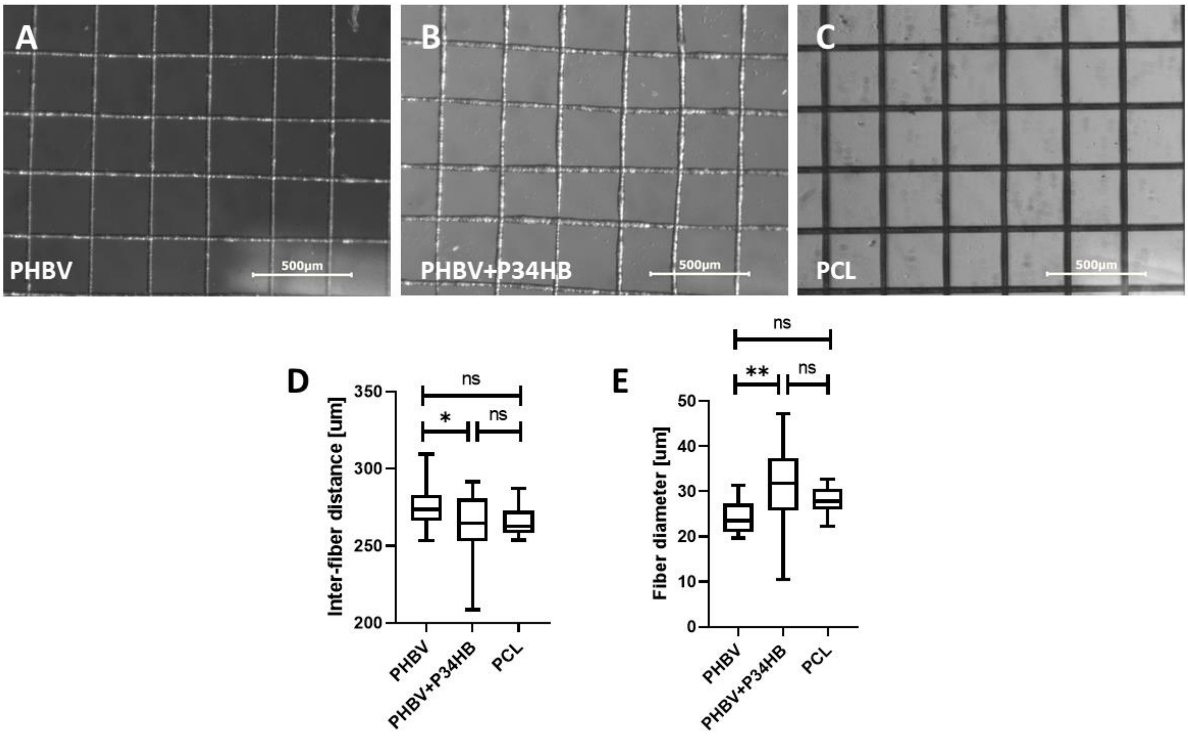

Figure S11. Brightfield microscopy images of PHA and PCL scaffolds for cell culture studies. A) PHBV, B) PHBV+P34HB, and C) PCL printed as square meshes; Comparison of D) inter-fiber distances and E) fiber diameters, measured for each type of square mesh scaffold. Data are presented as mean ± SD, n = 22. Significant statistical difference: *$p \leq 0.05$; **$p \leq 0.01$; ***$p \leq 0.001$; *ns* = non significant.

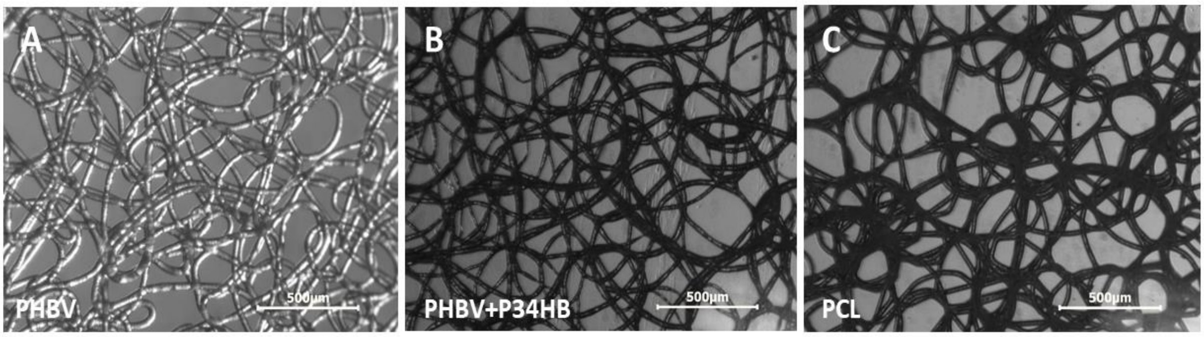

Figure S12. Brightfield microscopy images of PHA and PCL scaffolds for cell culture studies. A) PHBV, B) PHBV+P34HB, and C) PCL melt electrowritten into scaffolds with randomly deposited fibers.

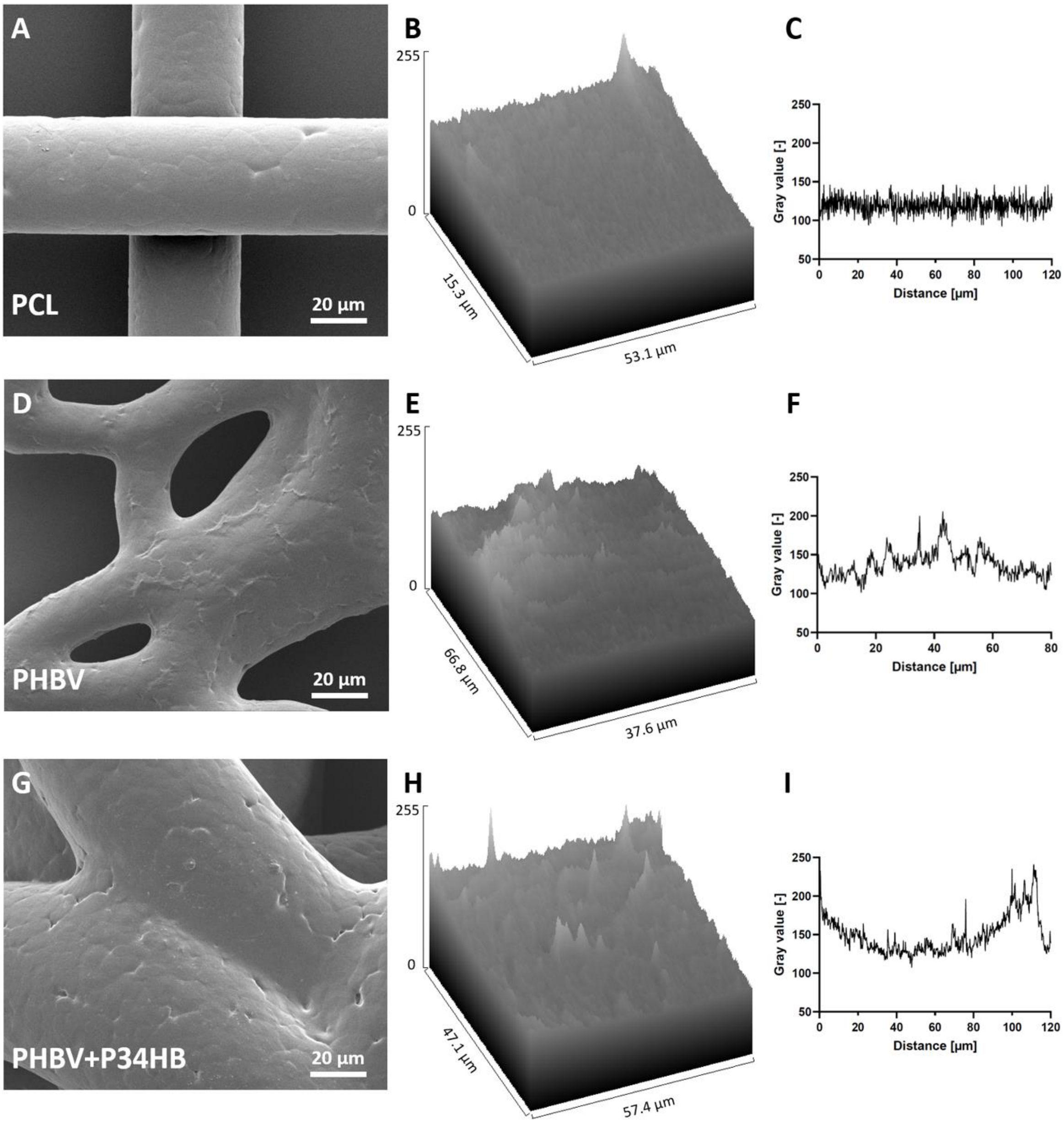

Figure S13. Surface roughness measurements. A) SEM image of PCL fibers with B) surface plot, and C) surface profile graph; D) SEM image of PHBV fibers with E) surface plot, and F) surface profile graph; G) SEM image of PPHBV+P34HB fibers with H) surface plot, and I) surface profile graph.

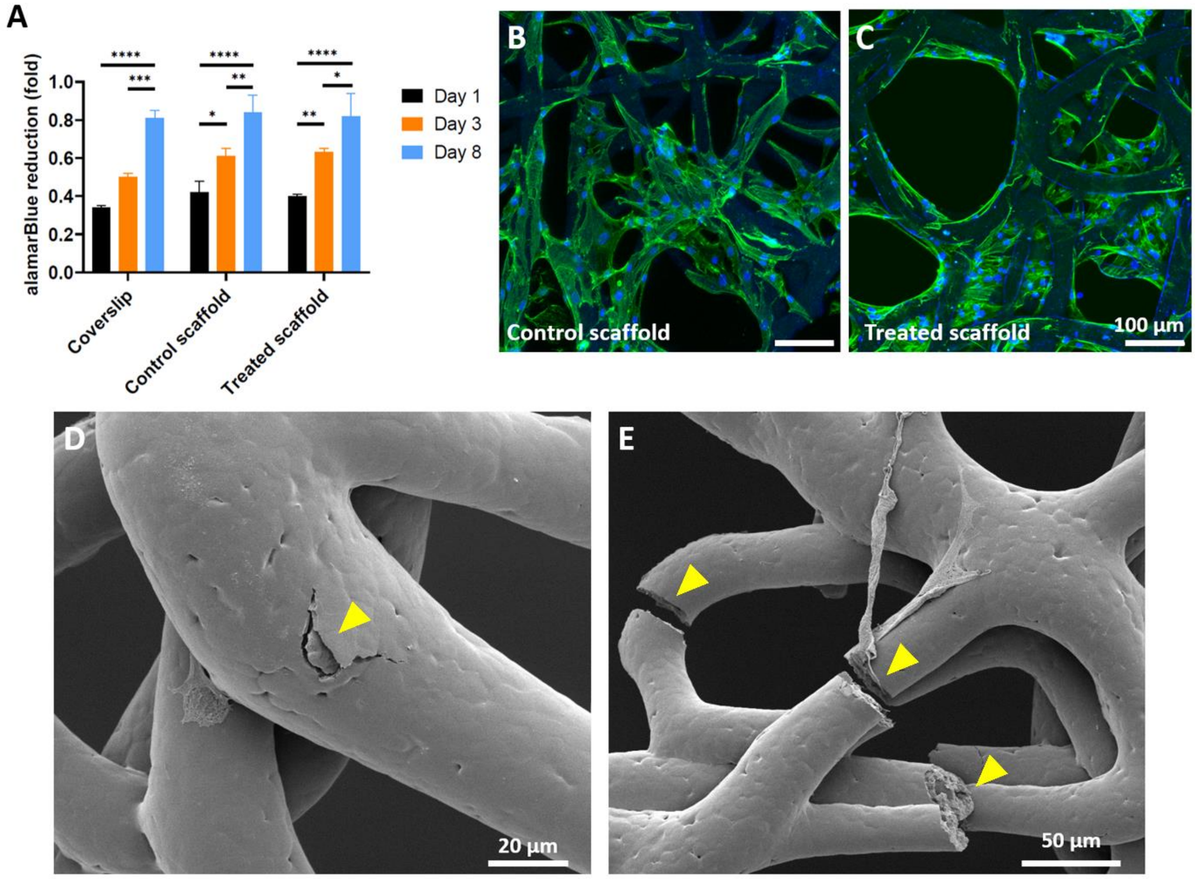

Figure S14. A) Cell metabolic activity assay conducted on HDF cells cultured on coverslips, control PHBV+P34HB random scaffolds and PHBV+P34HB random scaffolds exposed to the enzyme solution diluted with medium into 1:5 concentration. Data are presented as mean ± SD, $n = 3$. Significant statistical difference: *$p \leq 0.05$; **$p \leq 0.01$; ***$p \leq 0.001$; ****$p \leq 0.0001$; B, C) Immunofluorescence staining of HDF cells after 8 days of culture on B) control PHBV+P34HB random scaffold and C) enzymatically treated PHBV+P34HB random scaffold (1:5 enzyme dilution in medium). Dyes: DAPI (nucleus, blue), phalloidin (cytoskeleton, green). Scale bars: 100 µm. D-E) SEM images of enzymatically treated PHBV+P34HB random scaffolds (1:5 enzyme dilution in medium) and HDF cells grown on top of the fibers after 8 days of culture. Structural changes in the scaffolds are indicated with yellow arrows.

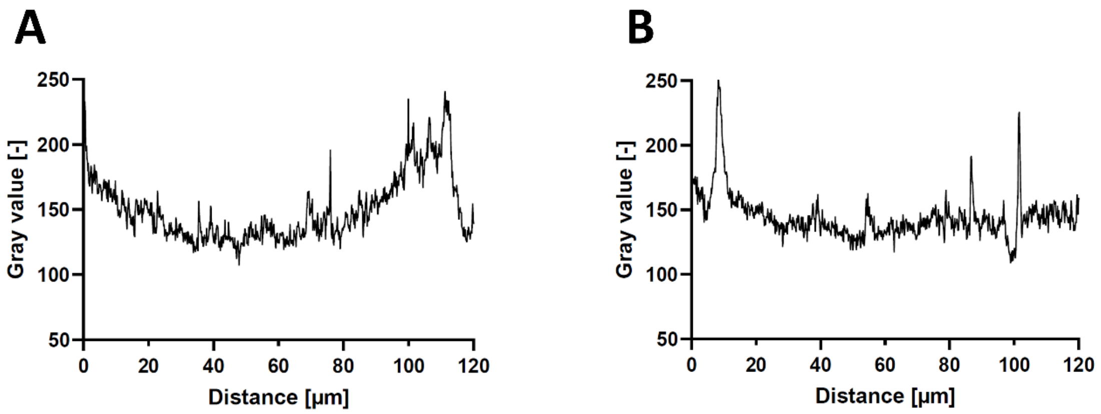

Figure S15. Comparison of surface profile graphs for A) the control PHBV+P34HB scaffold B) the enzymatically treated PHBV+P34HB scaffold after 8 days of culture with HDF cells.
